# Germline sequence variation within the ribosomal DNA is associated with human complex traits

**DOI:** 10.1016/j.xgen.2026.101213

**Published:** 2026-04-10

**Authors:** Francisco Rodriguez-Algarra, Elliott Whittaker, Maia Cooper, Sergey Koren, Maria R. Conte, Adam M. Phillippy, Faraz K. Mardakheh, David M. Evans, Vardhman K. Rakyan

**Affiliations:** 1 The Blizard Institute, School of Medicine and Dentistry, Queen Mary University of London, London, UK; 2 Centre for Epigenetics, Queen Mary University of London, London, UK; 3 Randall Centre for Cell and Molecular Biophysics, King’s College London, London, UK; 4 Department of Biochemistry, University of Oxford, Oxford, UK; 5 Genome Informatics Section, Center for Genomics and Data Science Research, National Human Genome Research Institute, National Institutes of Health, Bethesda, MD, USA; 6 Institute for Molecular Bioscience, The University of Queensland, Brisbane, QLD, Australia; 7 Frazer Institute, The University of Queensland, Brisbane, QLD, Australia; 8 MRC Integrative Epidemiology Unit, University of Bristol, Bristol, UK

**Keywords:** ribosomal DNA, ribosomal RNA, ribosome, germline variation, ribosome heterogeneity, UK Biobank, body size, expansion segments, human phenotypes, intragenomic frequencies

## Abstract

Ribosomal RNAs (rRNAs), essential components of the ribosome, are coded by the multi-copy ribosomal DNA (rDNA). Interestingly, rDNA displays substantial variation in all species, both as inter-individual differences in copy number (CN) and inter- and intragenomic sequence variation across copies (single-nucleotide variants [SNVs] and insertions/deletions [indels]). Whether germline rDNA sequence variation associates with human traits remains largely unknown. We here derive a stringently validated list of rDNA-associated SNVs and indels from UK Biobank whole-genome sequencing data, and we show that specific rDNA variants associate with human phenotypes independently of rDNA CN. Notably, variants within the 28S expansion segment 15L associate with body size traits. Variant combinations in the region present in actively translating ribosomes are predicted to alter the rRNA secondary structure. This represents the first large-scale association analysis of human traits with germline rDNA sequence variation, a largely ignored source of trait-relevant genetic variation to date.

## Highlights

• Estimation of ribosomal DNA (rDNA) variant frequencies in ∼500,000 individuals
• rDNA variant frequencies associate with multiple human complex traits
• Variants in expansion segment 15L (ES15L) associate with various body size traits
• ES15L variants are incorporated into translating ribosomes and alter rRNA structure

## Graphical abstract

**Figure:**
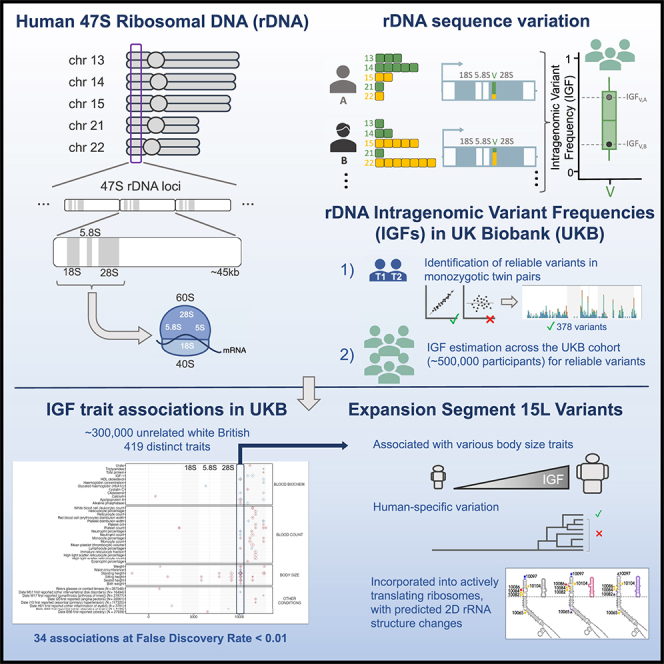

## Teaser

Rodriguez-Algarra et al. present an analysis of the prevalence of ribosomal DNA variants in UK Biobank participants, revealing associations with multiple human complex traits. Notably, a cluster of human-specific variants in expansion segment 15L, which are incorporated into ribosomes, associate with body size traits, such as height and weight.

## Introduction

**Figure 1:**
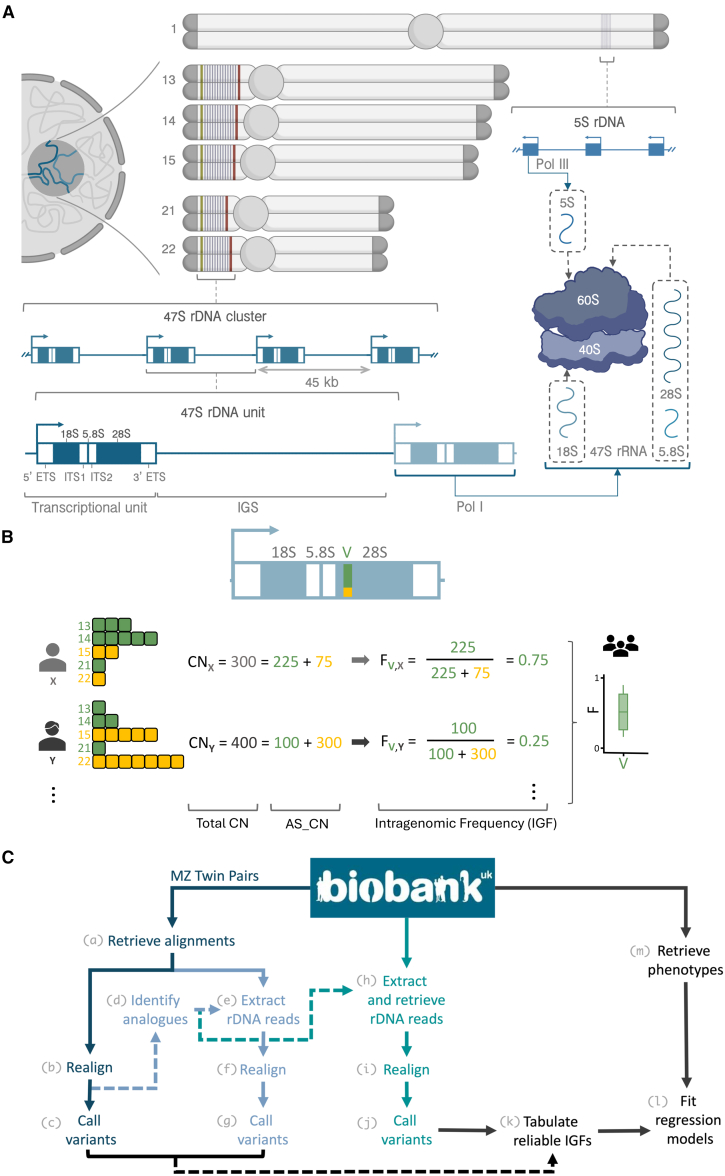
Ribosomal DNA and methodology schematics (A) Schematic representation of the human rDNA loci. Adapted from Rodriguez-Algarra et al.^13^ (B) Toy example of sequence variation in the rDNA. Given a variant position V within the 28S with two potential alleles G (yellow, reference) and A (green) and assuming for simplicity that the alleles at this position are chromosome specific (although from real short-read data, one cannot determine the chromosomal origin of observed rDNA variants), an individual X might harbor 225 of their total CN_X_ = 300 rDNA units with an A at that position (each square representing 25 rDNA units). In that case, their allele-specific CN for the variant V:G>A is AS_CN_V,X_ = 225, and their corresponding intragenomic variant frequency is thus F_V,X_ = 0.75. Another individual, Y, on the other hand, might only harbor an A in 100 of their total CN_Y_ = 400 rDNA units. Their allele-specific CN is hence AS_CN_V,Y_ = 100, and their corresponding intragenomic variant frequency is F_V,Y_ = 0.25. Across the population, the intragenomic variant frequencies at that position will likely follow a continuous distribution. (C) Validation and analysis pipelines employed in this study. To select a reliable set of rDNA variants to analyze, alignment files for White British UKB participants in MZ twin pairs were retrieved in full (a). These were then processed in two different ways. First, the full alignment files were realigned to diverse reference sequences (b). The results of these realignments were used both to compare the resulting variant frequencies (c) and to determine the location of rDNA analogue regions in Hg38 (d). The reliability of the variant calls themselves was determined by comparing the estimates within twin pairs. Second, reads from the pre-existing alignments mapping to regions of Hg38 identified as harboring rDNA analogue sequences were extracted (e) and realigned (f) in turn to determine the reliability of the extraction procedure (g). These validation steps then informed the procedure employed in the full cohort, where reads mapping to rDNA-analogue regions were extracted (h) prior to alignment (i). The resulting variant calls (j) were then tabulated (k) and regressed (l) against a range of UKB traits (m) to determine putative phenotypic associations with intragenomic variant frequencies.

The ribosome, as the cell’s translational apparatus, is one of the fundamental macromolecules of life (Figure 1A). In humans, the mature ribosome comprises ∼80 different proteins and 4 different ribosomal RNAs (rRNAs)—5S, 18S, 5.8S, and 28S—which are the most abundant RNA species in the cell. The high levels of rRNA expression are supported by the multi-copy nature of the ribosomal DNA (rDNA). Interestingly, due to elevated homologous recombination, rDNA copy number (CN) displays significant inter-individual variation (∼200–600 copies per human diploid genome).^1^^,^^2^ Furthermore, the different copies of rDNA are genetically variable, containing single-nucleotide variants (SNVs) and insertions/deletions (indels)^1^^,^^3^^,^^4^ (hereby collectively referred to as “variants”), demonstrating that sequence homogenization across different rDNA copies is only partial.^5^ Previous studies have found that these variants are located throughout the rDNA unit, with multiple variants possible at any given position, as distinct alternative alleles in SNVs or, more often, distinct indel sequences. This includes within rRNA subunits that are incorporated into the mature ribosome, leading to the intriguing possibility of rRNA-based ribosomal heterogeneity with phenotypic impact,^6^^,^^7^ such as differential translational outcomes,^8^^,^^9^^,^^10^ as previously reported for bacteria.^11^^,^^12^

But does naturally occurring genetic variation within human rDNA have a phenotypic impact? Until recently, this was difficult to answer because of the genetic complexity of rDNA clusters and because rDNA is either not represented on commercial microarray platforms or typically excluded in standard computational pipelines for analyzing sequencing data. This is now changing with the increasing availability of large-scale whole-genome sequencing (WGS) datasets and the development of novel computational approaches.^4^^,^^14^

To date, trait associations of human inter-individual rDNA genetic variation have been explored in the context of both somatic and germline variation.^13^ Somatic genetic variation of human rDNA has been noted in several biological contexts, most robustly in cancer,^15^^,^^16^ and also during aging,^17^ as well as in response to environmental insults,^18^^,^^19^ albeit in small-scale studies. Somatic variation includes both CN alterations and, in cancer, apparent differential expression of specific variant copies of the rDNA.^4^ However, in all these cases, rDNA genetic variation is most likely a downstream event, and it remains unclear whether somatic genetic variation of rDNA, including that which arises spontaneously, has any impact on trait outcomes.

On the other hand, trait-associated germline variation must either directly or indirectly influence the trait or disease in question. For germline rDNA genetic variation, we recently reported the first robust evidence for a genetic association between germline rDNA CN variation and a variety of human complex traits, including neutrophil counts and kidney disease in the UK Biobank (UKB)^14^ and body mass in a separate cohort.^20^ Importantly, we also established that rDNA CN variation is unlikely to be influenced by common genetic variation elsewhere in the genome.

The recent availability of WGS data from large, publicly accessible human biobank studies offers unprecedented opportunities to perform discovery analyses of the association between rDNA genetic variation and human traits. Here, we report an in-depth trait-association analysis of rDNA genetic variants in the UKB WGS data.^21^^,^^22^^,^^23^ We first derive a stringent list of rDNA-associated SNVs and indels that we validate in multiple ways. Using this list, we show that rDNA variants are associated with several human traits, most notably with body-size-related traits. 2D structural modeling reveals that specific sets of variants are likely affecting the structure of expansion segments (ESs) of the ribosome. Our work constitutes the first large-scale association analysis of human traits with germline sequence variation in the rDNA, which represents a source of human complex trait-relevant germline genetic variation that has thus far been ignored.

## Results

### Selection of rDNA sequence variants

Due to the hundreds of rDNA units that exist in the human genome, SNVs and indels in the rDNA theoretically exist in any relative proportion from 0% to 100% within an individual (Figure 1B). For example, if we consider variant 7980:G>A in an individual with a total rDNA CN of 400, of which 300 harbor Gs (the reference allele) and the rest As, the intragenomic variant frequency (IGF) at 7980:G>A in this individual would be 0.25 (i.e., 25%, and the value used in further analyses). This property of rDNA variation impedes the use of conventional variant-calling tools, possibly contributing to the discrepancies among the sets of variants reported in previous studies.^1^^,^^3^^,^^4^ Since our main goal is to determine whether any rDNA sequence variation within the 47S transcriptional unit associates with human phenotypes and not necessarily to determine how many variant positions exist in the region, we devoted substantial efforts to establish a set of reliable rDNA variants in the UKB cohort. We therefore first focused on establishing a pipeline for deriving stringent germline rDNA variants that could be used for further analysis of human WGS data.

Because of their genetically identical nature, monozygotic (MZ) twin pairs allow for robust validation of an rDNA variant identification pipeline, as intragenomic frequency estimates for germline rDNA variants should, in principle, strongly correlate within an MZ twin pair. Indeed, our previous analysis of the UKB showed that MZ twins display very high intra-pair rDNA CN correlations.^14^ For the current analyses, we focused on WGS data from all 49 White British (WB) MZ twin pairs (*n* = 98 individuals) where both individuals had been sequenced in the same center and release (since sequencing center can impact rDNA analyses^14^). These pairs were then used to validate the core components of the pipeline: reference assembly for realignment, the quality of the variant calls themselves, and the effect of extracting reads mapping to rDNA-analogue regions from the pre-existing alignments (Figure 1C).

### Choice of reference assembly for realignment of rDNA reads

**Figure 2:**
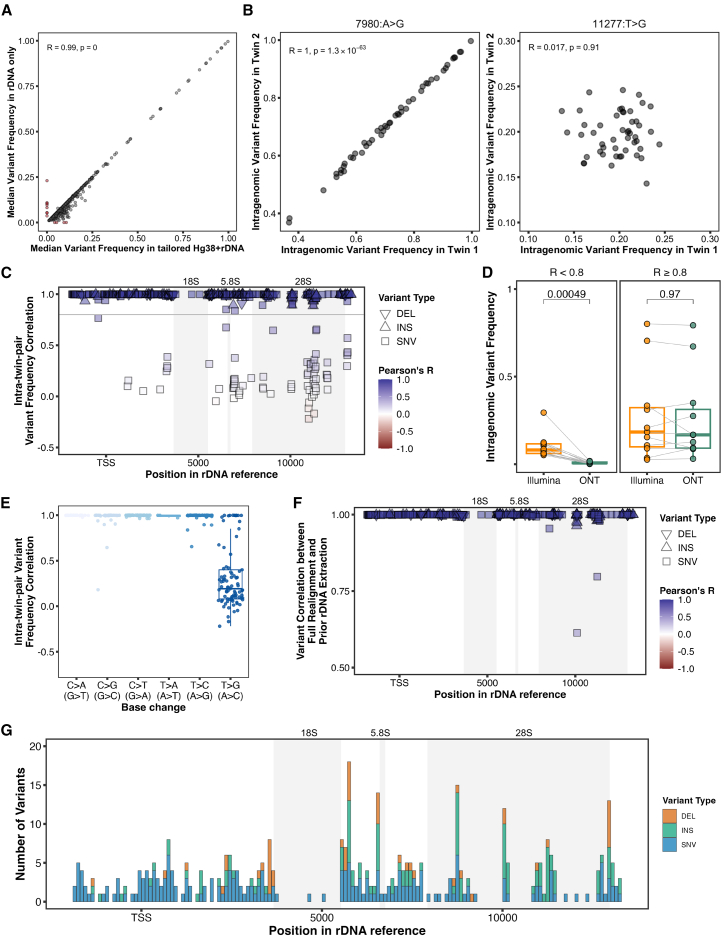
Estimation and selection of rDNA variants for analysis (A) Comparison between the median frequency for each identified variant in the rDNA transcriptional unit (Pearson’s R, *n* = 513 variants) estimated from realignments of UKB MZ twin samples to the full tailored Hg38+rDNA consensus assembly and a looped GenBank: KY962518.1 rDNA reference. Red dots indicate variants that were not detected at any frequency in only one of the approaches. (B) Examples of intragenomic variant frequencies comparisons in MZ twins (Pearson’s R, *n* = 49 twin pairs). The left panel shows an rDNA variant with high intra-twin-pair correlation, whereas the right panel shows a variant with extremely low correlation. (C) Intra-twin-pair correlation (Pearson’s R) in estimated variant frequencies across the rDNA transcriptional unit. The horizontal line indicates the chosen R = 0.8 threshold. Here and in (F) and (G), the gray-shaded areas represent, from left to right, the 18S, 5.8S, and 28S subunits. (D) Comparison between the 28S variant frequencies (paired Wilcoxon’s rank sum, *n*_R<0.8_ = 12, *n*_R≥0.8_ = 11) detected from Illumina short reads and Oxford Nanopore (ONT) long reads for HG00127, a GBR participant from the 1000 Genomes Project, split by the intra-twin-pair correlation of each variant obtained on UKB MZ twins. The *p* value for Wilcoxon ranked test for the difference in means is indicated in each panel. (E) For the SNVs in Figure 2C, the relationship between the intra-twin-pair correlation (Pearson’s R) in estimated variant frequencies and the change in nucleotide corresponding to the specific variant. The changes in parentheses indicate that complementary changes were grouped together. (F) Correlation (Pearson’s R) between variant frequencies across the rDNA transcriptional unit estimated from full library realignments and the prior extraction of reads mapping to rDNA-analogue regions. (G) Positional distribution across the human rDNA transcriptional unit of variants selected for further analysis.

Typically, WGS-based human rDNA sequence analysis involves aligning the entire dataset to a tailored reference assembly that appends a single rDNA unit to the entire consensus sequence for the rest of the genome (e.g., Hg38).^14^^,^^20^^,^^24^^,^^25^^,^^26^ This approach is designed to reduce the likelihood that spurious alignments to the rDNA from reads arising from elsewhere in the genome will affect the rDNA results. Such an approach, however, is problematic for a dataset the size of the UKB. Alternatively, one can align solely to an rDNA unit or rRNA gene sequence consensus,^1^^,^^3^^,^^4^ greatly reducing the computational burden, but at the risk of less reliable variant calls. We wondered whether the increase in efficiency may outweigh the potential noise introduced in the calls, thereby allowing us to process the UKB WGS data. We therefore realigned the full WGS data for the 98 UKB individuals (49 WB MZ twin pairs) to a tailored whole-genome plus rDNA reference^14^^,^^20^^,^^25^ and to a sole looped rDNA consensus. As Figure 2A shows, differences in the intragenomic variant frequencies from the rDNA transcriptional unit derived from these two methods only arise at the lower range of frequencies (Pearson’s R = 0.99, *p* = 0). We observed similar results when employing other publicly available whole-genome plus rDNA references^26^ (Figure S1). We thus decided to introduce a pragmatic variant-filtering step, retaining only variants that appeared in at least one individual among the twin pairs in high-confidence calls (see STAR Methods) with intragenomic frequency between 10% and 90%. 457 variants across the rDNA transcriptional unit satisfied this requirement (note that we did not consider the intergenic spacer [IGS] in this study).

### Intra-twin pair frequency comparisons reveal sequencing artifacts

Next, for each of the 457 variants, we calculated the correlation between the IGF estimates for the two individuals of each MZ twin pair. For most variants, the Pearson’s correlation value is approximately 1 (e.g., Figure 2B, left). However, for 77 out of the 457 variants (16.8%), the observed correlation was substantially lower (e.g., Figure 2B, right), with apparent clusters of diverging variants throughout the rDNA transcriptional unit (Figure 2C). Both concordant and divergent variants appear even at low average IGF ranges (Figure S2), with the concordant set showing a higher prevalence of low IGF variants but higher average read depth (Figure S3), suggesting concordant variants tend to accumulate in regions of higher mappability. A comparison of matched short-read Illumina and long-read Oxford Nanopore (ONT) data for a 1000 Genomes Project (1kGP) GBR (British in England and Scotland) individual revealed variants with R < 0.8 in UKB MZ twins are virtually undetectable in ONT data, but the two technologies agree for variants with R ≥ 0.8 (Figures 2D and S4). This strongly suggests that discrepant frequencies in MZ pairs are technical in origin. Moreover, 74 out of 77 R < 0.8 SNVs are T>G (or, equivalently, for the other strand, A>C; Figure 2E), suggesting that at these positions, the Illumina base calls for actual Ts are reported as Gs (and, equivalently, As reported as Cs). Recent reports have also observed substantially higher rates of transversion errors in Illumina reads than on other platforms,^27^ particularly in T>G and A>C substitutions.^28^ The two-channel sequencing by synthesis (SBS) technology employed in the Illumina NovaSeq 6000 instrument might explain this effect, coupled with the high GC content of specific rDNA regions. IGFs and read depths for the 3 non-T>G/A>C discordant variants are shown in Figure S5. We discarded all 77 R < 0.8 variants, leaving 380 variants for further consideration.

### Focusing on Hg38 rDNA-analogue regions minimally impacts rDNA variant frequencies

rDNA analogues were identified as those regions within the Hg38 reference sequence where no explicit rDNA unit appears but sequencing reads originating from the rDNA unit still map. These regions largely consist of full or partial unannotated rDNA units in chr21 or “unplaced contigs” of the assembly. In this case, our region of interest extends beyond the highly conserved 18S we employed for our rDNA CN analysis in the UKB^14^ to the entire transcriptional unit (here defined as spanning from position −1870, upstream of the transcriptional start site [TSS], to the end of the 3′ external transcribed spacer [ETS]). To further reduce the computational burden of our analysis, we considered extracting reads mapping to rDNA-analogue regions from the pre-aligned WGS libraries prior to rDNA realignment. We thus aimed to locate and validate suitable analogue regions. We generated synthetic Illumina reads from the rDNA consensus reference and aligned them to the Hg38 assembly. We also determined where reads mapping to the rDNA transcriptional unit in the realigned MZ twin data above originally mapped in Hg38. Both approaches coincide for the rDNA transcriptional unit, reporting virtually all synthetic reads and over 98% of the real reads mapping to the same Hg38 windows neighboring the 18S rDNA analogues we previously employed for the rDNA CN analysis (Figure S6).^14^ Furthermore, comparing the IGFs obtained from full realignments with those from prior extraction of these putative analogue regions in the MZ twin data confirmed the validity of this latter approach, with only two variants having a Pearson’s R < 0.8 (Figure 2F). In both cases, the reduced correlation appears to arise due to the variant not being detected in a small subset of individuals rather than from systematic biases in the estimates that could suggest missing analogue regions (Figure S7). Regardless, we only retained variants with an R > 0.8 between full realignments and prior analogue extraction, resulting in 378 sequence variants for further analysis (Table S1). To note, this set of variants does not represent the full landscape of rDNA variation but rather a high-quality set for further trait-association analyses.

The 378 variants are located across 317 distinct positions between −1848 (upstream of the TSS) and 13294 (within the 3′ ETS) of the GenBank: KY962518.1 rDNA consensus reference.^29^ In particular, they correspond to 217 SNVs and 161 indels (42.6%), 48 of which are reported as deletions and 113 as insertions when compared to said reference. This represents a higher proportion of indels than previously reported from short-read data (usually below 30%^3^^,^^4^^,^^30^) but substantially lower than estimates from long-read data (∼75%^4^), suggesting the stringent set may alleviate the biases of each sequencing approach. We observed a higher density of variation throughout the promoter and spacer regions (3.34 variants in the stringent set per 100 bp of sequence) compared with the rRNA coding subunits (1.51 variants per 100 bp), with 28S (2.04 variants per 100 bp) harboring substantially more variation than 18S or 5.8S (0.16 and 0.64 variants per 100 bp, respectively; Figure 2G). For some variants, virtually all twin pairs harbor at least two alleles, whereas for others, most pairs show no variation whatsoever, and only a few pairs display some degree of bi-allelism, with the reference allele usually clearly dominating (Figure S8). For example, the previously described variant located 60 bp into the 28S sequence^31^ (here reported as 7980:A>G, shown in Figure 2B) belongs to the first group, whereas the small subset of detected 18S variants belongs to the latter (Figure S9).

### rDNA variant frequencies associate with human traits in the UKB

For each of the 378 variants, we then asked if their IGF associates with phenotypic traits. We therefore obtained frequency estimates for 490,398 UKB participants using LoFreq^32^ but only employed a set of 297,010 unrelated WB individuals in association tests. Although these estimates might suffer from reference-biased mapping, potentially favoring the allele present in the reference sequence due to preferential mapping,^33^ they are suitable proxies for the real underlying IGFs. Tests were then conducted independently for each variant (a necessary simplification, since variant frequencies will often correlate) using PHESANT,^34^ regressing phenotypic traits on rDNA IGFs (valued between 0 and 1, a procedure analogous to what we previously employed for rDNA CN^14^). We limited the set of considered phenotypes to 419 distinct traits for which PHESANT provided valid output. This reduced set excludes, among others, fields related to cancer (such as diagnoses and histology results), as analysis of existing large, devoted cohorts that include both germline and tumor WGS data will provide more powerful insights into the potential role of rDNA sequence variation in cancer.

**Figure 3:**
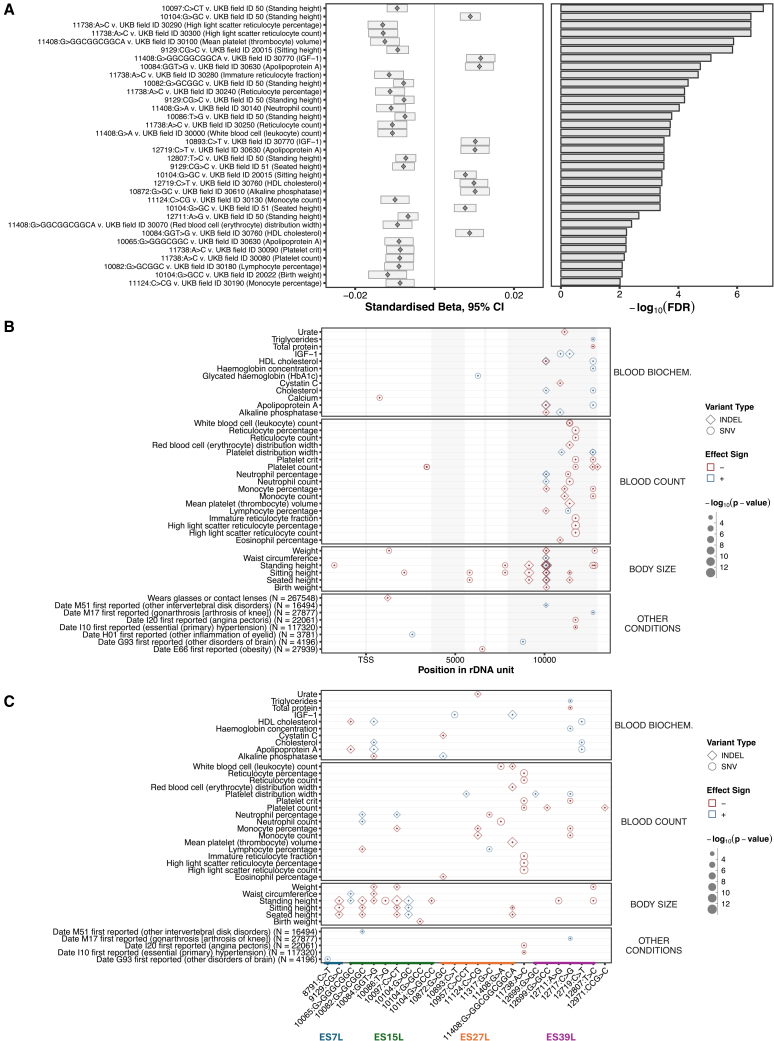
Association between rDNA intragenomic frequencies and traits in the UKB (A) Effect size (left) and significance level (right) of associations between intragenomic variant frequencies and UKB phenotypes at a global FDR < 0.01, estimated from unrelated WB individuals. (B) Positional distribution of phenotypic associations across the rDNA transcriptional unit with a variant-level FDR < 0.01 (37 distinct variants). (C) Phenotypic associations with a variant-level FDR < 0.01 within the 28S (a subset of 25 variants from B). The corresponding expansion segments are indicated.

The regressions yielded 34 associations at a global false discovery rate (FDR) < 0.01 (Figure 3A; Table S2), corresponding to 17 distinct variants (Figure S10), all located within the 28S. Of these, 26 have *p* values below the Bonferroni threshold of 3.3 × 10^−7^ for the 151,317 considered tests. The top two FDR-significant associations both correspond to “standing height” for variants located less than 10 bp apart: 10097:C>CT is negatively associated with the trait (*p* = 4.98 × 10^−13^), whereas 10104:G>GC is positively associated (*p* = 4.27 × 10^−12^). In fact, several neighboring variants also associate with height and/or other body-size- or growth-related phenotypes, particularly when considering variant-level FDR significance (Figure 3B; see STAR Methods). A single cluster of variants around position 10100 associates not only with diverse body size measurements (including “weight,” “waist circumference,” and “birth weight,” aside from different versions of height) but also with “cholesterol,” “apolipoprotein A,” and “HDL cholesterol”—closely associated with weight—as well as “other intervertebral disk disorders”—which could relate to height. A recent study reports similar observations.^35^ Interestingly, the difference in height between the highest and lowest deciles of height-associated rDNA variant frequencies (3–4 mm) is not dissimilar to the effect sizes of the most strongly associated common genetic variants for height.^36^

Closer inspection revealed that the cluster around 10100 is, in fact, formed by 8 distinct variants at 6 different positions within a span of fewer than 40 bp (Figure 3C). These all fall within expansion segment 15 of the large ribosomal subunit (ES15L). In fact, the vast majority of these variants with putative phenotypic associations fall within ESs. The exceptions are 12971:CCG>C, which coincides with the 3′ end of 28S, and 11738:A>C, whose association with multiple cardiovascular- and myeloid-related phenotypes will be important to study in the future.

### rDNA CN does not impact variant-trait associations

**Figure 4:**
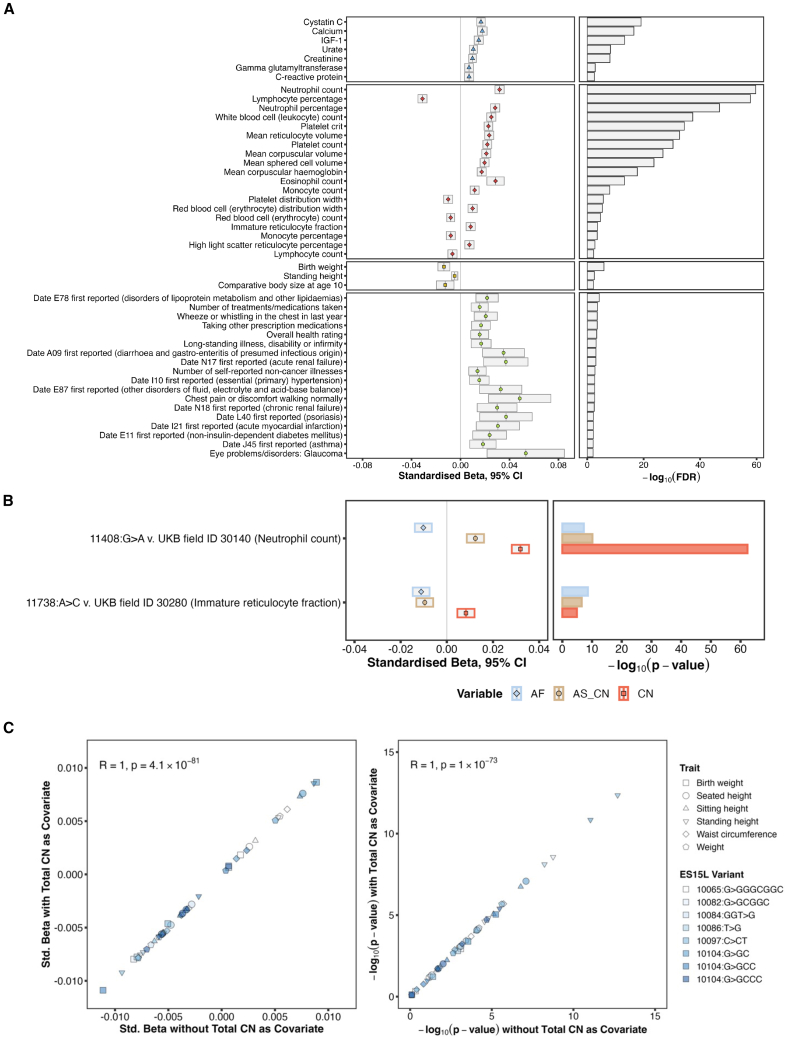
Impact of rDNA CN on trait associations (A) Effect size (left) and significance level (right) of phenotypic associations with 18S Ratio (a proxy for total rDNA CN) in UKB White British participants, combining both sequencing releases. Expanded from Rodriguez-Algarra et al.^14^ (B) Comparison between effect sizes (left) and significance levels (right) obtained for intragenomic variant frequency (AF) and allele-specific CN (AS_CN) in two combinations of variant and phenotype reaching statistical significance in all cases, plus the results for the association between the corresponding phenotypes and total rDNA CN for reference. (C) Comparison between effect sizes (left) and significance levels (right) obtained for all phenotype-associated variants in ES15L with and without including total rDNA CN as a covariate in the regression models for body size phenotypes (Pearson’s R, *n* = 48).

Interestingly, many rDNA-variant-associated traits are similar to those we previously found associated with total rDNA CN.^14^^,^^20^ We therefore wondered if rDNA CN impacts rDNA variant associations. We first conducted a more powerful analysis of rDNA CN trait associations in the UKB, including all 297,010 unrelated WB individuals in a single analysis (Figure 4A; Table S3). All the previously identified associations remained highly significant. New associations included three measurements in the body size category: birth weight, standing height, and “comparative body size at age 10,” further extending the overlap with allelic associations.

We then investigated if the absolute number of rDNA copies harboring a specific variant allele (i.e., a particular version of the DNA sequence at a variant position) associates with phenotype (see Figure 1B). For example, given two individuals (say, X and Y) with different total number of rDNA copies (say, CN_x_ = 400 and CN_y_ = 200) and different intragenomic frequencies at a given variant (say, F_x_ = 0.25 and F_y_ = 0.5), they might experience the same contribution to a particular phenotypic outcome because their cells harbor the exact same number of rDNA copies with a variant allele (in this case, 100). We call this an allele-specific CN (AS_CN), which can be calculated for each variant of interest as the product of the IGF and total rDNA CN. Comparing the effect sizes obtained in linear regression models using variant frequency as an independent variable (Figure S11) with those including AS_CN (Figure S12) clearly shows an increase in the effect magnitudes across the unit for those phenotypes already shown to associate with total rDNA CN (Figures S13 and 4A). In general, the effect of the total number of copies is greater than that of the specific allele. This is clearly seen in variants such as 11408:G>A (Figure 4B, top), where the association between its IGF and neutrophil counts is significantly negative (β = −0.0102 [−0.0139 to −0.00650], *p* = 5.84 × 10^−8^) but, when calculating the association with its AS_CN, the effect becomes positive (β = 0.0124 [0.00865 to 0.0161], *p* = 7.30 × 10^−11^), aligning with the effect of the total number of copies. When the effect of total CN is still opposed to the allelic effect but with smaller intensity, such as the case of the “immature reticulocyte fraction” in 11738:A>C (Figure 4B, bottom), the effect remains significantly negative in both associations with frequency (β = −0.0111 [−0.0148 to −0.00747], *p* = 2.48 × 10^−9^) and AS_CN (β = −0.00946 [−0.0132 to −0.00590], *p* = 3.00 × 10^−7^), but trending toward the direction of the total CN association.

When both allelic and CN components are considered, CN effects tend to dominate. However, when we calculated the difference in the effect sizes obtained both with and without CN as a covariate, we found negligible differences in most variant-trait combinations, with only one variant (11317:G>C) showing any meaningful change (Figures S14 and S15). In the particular case of the associations between body size measurements and the frequency of ES15L variants highlighted above, we see virtually no change in either effect sizes or significance levels (Figure 4C). Therefore, although rDNA CN and sequence variants might influence similar traits, their effects are independent.

### Key genomic properties of trait-associated ES15L variants

ESs are inserted regions within the eukaryotic rRNAs, greatly increasing their size compared with the prokaryotic counterparts. They tend to locate as protrusions on the surface of the mature ribosomes (Figure S16). These regions harbor most rRNA variation across and within species, but their function is still poorly understood.^4^^,^^37^ It is believed that they may contribute to modulating translation by binding ribosome-associated proteins.^38^ Moreover, it has been noted that 28S ESs partly resemble mRNA sequences, particularly ES15L, which also appears to have been greatly expanded in mammals, particularly in hominids.^39^ Similar to what others have previously reported,^4^ multiple variants we observed in ES15L (particularly those at positions 10065, 10082, 10084, and 10086) tend to affect the number of GGC tandem repeats in the region.

**Figure 5:**
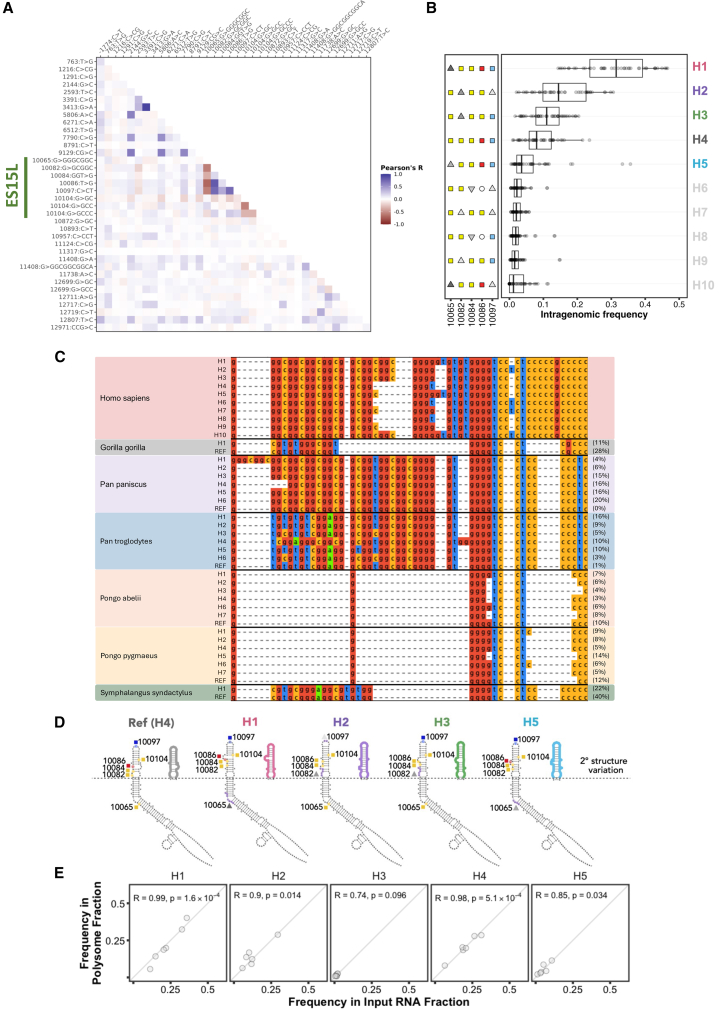
Genomic properties of ES15L variants (A) Heatmap of correlations between intragenomic variant frequencies across pairs of variants with variant-level FDR < 0.01 associations (Figure 3B), with variants located within ES15L marked. (B) Ten most frequent combinations of alleles at a subset of ES15L variants in MZ twins sequenced by deCODE in the second sequencing release. Squares represent single nucleotides (C is blue, G is yellow, and T is red), triangles represent indels (upward triangles represent insertions and downward triangles represent deletions, with their darkness representing the size of the indel itself), and circles represent absent positions (due to a prior deletion spanning them). The combination labeled as H4 coincides with the sequence present in the GenBank: KY962518.1 rDNA consensus sequence. (C) Zoom in on the multi-alignment of primate ES15L sequences employed to generate the phylogenetic tree in Figure S17 around the human trait-associated variant combinations in Figure 5B. For all non-human entries, the percentage on the right indicates the prevalence at which the corresponding sequence was observed in the samples employed for their identification. Note that some sequences appear identical in this region (such as both from *Gorilla gorilla*), but they differ elsewhere in ES15L. (D) Predicted secondary structure RNA models of the 5 most frequent combinations of alleles at a subset of ES15L variants. Symbols and colors indicate SNVs and indels as in (B). Changes to secondary structure are observed above the dashed line. (E) Comparison (Pearson’s R, *n* = 6) between frequencies of the five most common combinations of ES15L variants (see B) obtained on input RNA and polysome fractions from GBR 1kGP participants.

As noted before in smaller cohorts,^4^ the correlation between 28S IGFs in the UKB is substantially higher within ESs, particularly within ES15L (Figure 5A). ES15L variants were not included in previous attempts to build 28S haplotypes (or “morphs”), but the tight correlation between the IGFs within the region suggests they might form local haplotypes. Preliminary analyses suggested that might be the case, particularly when considering the multiple variants at position 10104 separately, with some combination of alleles at positions 10065, 10082, 10084, 10086, and 10097 appearing in a median of around 30% of each individual’s rDNA units (Figure 5B). Multiple other combinations seem to form 15% or fewer of the rDNA units, leading to morph profiles that are substantially less clearly defined than what was previously observed in inbred mice.^25^ Nevertheless, in the future, it might be worth exploring phenotypic associations with the intragenomic prevalence of these variant combinations instead of each one separately.

Given the apparent expansion of ES15L in hominids, we wondered whether the trait-associated variants identified in the region were preserved across the ape lineage. To this end, we obtained HiFi sequencing reads from the recently constructed complete ape genomes for bonobo (*Pan paniscus*), chimpanzee (*Pan troglodytes*), western gorilla (*Gorilla gorilla*), Bornean orangutan (*Pongo pygmaeus*), Sumatran orangutan (*Pongo abelii*), and siamang (*Symphalangus syndactylus*).^40^ From these, we identified reads spanning the ES15L, according to the highly conserved flanking regions, and clustered them for each species based on their sequence, retaining morphs appearing in at least 5% of the reads. We then multi-aligned the identified morphs across all species, including the equivalent human sequences in Figure 5B, from which we derived a phylogenetic tree (Figure S17). Except for the two closely related species of orangutan (diverged less than 500,000 years ago^41^), where morphs from *Pongo pygmaeus* and *Pongo abelii* often cluster together, all other species form separate clusters. Human sequences, in particular, are clearly separate (94% bootstrap support), suggesting that the variation identified in the region is species specific (Figure 5C).

Finally, comparisons of 2D structural models of the top 5 most frequent human ES15L haplotypes suggest that the rRNA conformation changes across observed combinations of variants in the region (Figures 5D and S18). This could impact how the ES protrudes out of the ribosome’s surface and influence protein and/or mRNA binding, potentially affecting human trait outcomes via differential modulation of translation. For this mechanism to be feasible, however, the ES15L variants must be expressed and incorporated into actively translating ribosomes. Analysis of publicly available RNA sequencing (RNA-seq) data^42^ shows that the expression levels of ES15L variants are similar to their corresponding IGFs in DNA (Figure S19). In addition, we generated input RNA and polysome-seq libraries from the lymphoblastoid cell lines (LCLs) of 6 GBR 1kGP participants (Figure S20). ES15L variants in our generated input RNA fraction show congruent expression frequencies with publicly available RNA-seq data^42^ (Figure S21), with the most salient differences occurring in the most lowly expressed variants (10084:GGT>G and 10104:G>GCCC). We then grouped variants per read into the same combinations from Figure 5B and compared their frequencies with WGS data from the same participants (Figure S22). This revealed that, similar to what we previously observed in inbred mice^25^ and has recently been reported in CHM13,^43^ allele-specific silencing might exist, with H3 appearing largely silent in most samples and H4 conversely overexpressed (Figure S23). Epigenetic or transcriptomic data in the UKB could thus help refine the identified phenotypic associations. Frequencies in input RNA and polysome fractions of our own generated data, on the other hand, match closely (Figure 5E), confirming that ES15L rRNA variants are incorporated into actively translated ribosomes at rates similar to their expression levels,^4^^,^^25^ thus potentially influencing ribosome function.

## Discussion

Here, using a stringent variant identification strategy, we have shown that germline SNVs and indels within the human rDNA, a region that has largely been ignored in human association studies to date, are associated with a variety of complex traits, especially in regions with species-specific variation. A key challenge now is to elucidate the mechanistic details of how rDNA genetic variants influence genome function. Inter-individual genetic variation of SNVs and indels in the rDNA has been observed in all organisms examined in this regard to date, and in bacteria, it has already been demonstrated that rDNA genetic variants influence translational outcomes.^11^^,^^12^ Could similar mechanisms be operating in humans? It is now recognized that the ribosome displays extensive compositional heterogeneity within an organism.^44^ Potentially, this could dynamically influence translational activity during normal development and disease.^10^ However, to date, most work on ribosomal heterogeneity has focused on proteins that associate with the ribosome, and the impact of rDNA (rRNA) genetic variation has been much less studied. It could be postulated that the incorporation of genetically different rRNAs could impact the binding of either proteins or even mRNAs. In the latter case, intriguingly, it has been observed that ESs match the 5′ ends of mRNAs,^39^ although it remains to be determined if and how this influences ribosomal function. Regardless, our results provide an important starting point for further investigation into the role of the enigmatic ESs. Of course, the rDNA genetic variation might impact other aspects of ribogenesis or ribosome function, including rRNA modifications.^45^^,^^46^

An obvious question is why rDNA variants are associated with specific traits, given that ribosomal variants could potentially modulate the translation of any expressed transcript. First, it could be a consequence of statistical power—traits such as blood counts, height, and weight are quantitative traits that can be measured accurately and are available for nearly all UKB participants. Nevertheless, it is interesting to note that in ribosomopathies—diseases caused by mutations in ribosomal component proteins or other proteins involved in ribosome biogenesis—effects on hematopoiesis are a defining feature.^47^ With respect to growth phenotypes, we have previously shown that a specific rDNA variant in the mouse is differentially methylated in response to *in utero* protein restriction, and this is associated with reduced weaning weight.^48^ It is also interesting to note that in actively growing *E. coli* species*,* the relative and absolute expression levels of genetically different rDNA operons can vary with growth conditions, with differential effects on translational outcomes.^11^ It is intriguing that many associations are shared between rDNA CN and rDNA variants, but the associations are apparently not influenced by each other. At this stage, it is difficult to conclusively determine why this is without a rigorous mechanistic dissection of how rDNA CN and variants impact molecular outcomes, which would require suitable tools for genetically editing mammalian rDNA.

### Limitations of the study

First, our stringent selection approach based on just 49 MZ twin pairs means that we have only considered common rDNA variants. WGS data from larger collections of MZ twins or much larger datasets that provide both short- and long-read sequencing data will allow robust identification of additional rDNA variants. Second, exploring additional biobank-level datasets will be required to ascertain the impact of rDNA variation in other ancestries and diseases. Third, integrating epigenetic information and rRNA profiling will provide important insights into the impact of rDNA genetic variation on molecular outcomes, especially if profiled in a range of tissues or developmental stages. Fourth, single-cell and/or single-molecule analyses will allow more direct associations between rDNA variation and ribosomal output and provide a measure of somatic heterogeneity of rDNA variation, should it exist. Finally, the most powerful approach would be the ability to genetically modify rDNA genetic variation and combine this with a range of molecular and structural studies. If such an approach could be successfully developed for the rDNA, it would also permit relevant follow-up studies of polysome-seq data, where one could measure protein levels after modification.

Understanding the relationship between genotype and phenotype is one of the principal challenges in biology. Despite the highly conserved nature of rDNA, we have shown that germline genetic variation in this region has phenotypic consequences. This variation is not captured by previous studies and represents an important new avenue to better explain how genotype influences phenotype.

## Resource availability

### Lead contact

Further information and requests should be directed to and will be fulfilled by the lead contact, Vardhman K. Rakyan (v.rakyan@qmul.ac.uk).

### Materials availability

This study did not generate new unique reagents.

### Data and code availability

Analysis scripts employed in this study are available at https://doi.org/10.5281/zenodo.18886743. All generated sequencing data are available at SRA BioProject ID PRJNA1405551. Variation estimates will be available as a return for UKB application ID 83271.

## Acknowledgments

F.R.-A. and V.K.R. were supported by grants from the Biotechnology and Biological Sciences Research Council (BBSRC; BB/R00675X/1). F.R.-A. was additionally supported by a Barts Charity seed grant (G-002983). E.W. was supported by a Barts Charity project grant (G-002663). M.C. was supported by a BBSRC London Interdisciplinary Biosciences Consortium (LIDo DTP) PhD studentship. S.K. and A.M.P. were supported by the Intramural Research Program of the US National Human Genome Research Institute, National Institutes of Health (NIH). The contributions of NIH authors are considered works of the US government. The findings and conclusions presented in this paper are those of the authors and do not necessarily reflect the views of the NIH or the US Department of Health and Human Services. F.K.M. was supported by a BBSRC grant (BB/X007820/1) and a Cancer Research UK Program Foundation Award (DRCPFA-Nov24/100005). D.M.E. was supported by an NHMRC Investigator Grant (APP2017942). We acknowledge the assistance of the ITS Research team at Queen Mary University of London (QMUL). This research utilized QMUL’s Apocrita HPC facility, supported by QMUL Research-IT (https://doi.org/10.5281/zenodo.438045).

## Author contributions

Conceptualization, F.R.-A. and V.K.R.; data curation, F.R.-A. and S.K.; formal analysis, F.R.-A., M.C., and S.K.; funding acquisition, V.K.R., D.M.E., F.K.M., A.M.P., and M.R.C.; investigation, F.R.-A., E.W., M.C., and S.K.; methodology, F.R.-A., E.W., S.K., A.M.P., F.K.M., D.M.E., and V.K.R.; project administration, V.K.R.; software, F.R.-A. and S.K.; supervision, V.K.R., D.M.E., F.K.M., A.M.P., and M.R.C.; visualization, F.R.-A. and M.C.; writing – original draft, F.R.-A. and V.K.R.; writing – review & editing, F.R.-A., E.W., M.C., S.K., M.R.C., A.M.P., F.K.M., D.M.E., and V.K.R.

## Declaration of interests

The authors declare no competing interests.

## STAR★Methods

### Key resources table

**Table:** 

REAGENT or RESOURCE	SOURCE	IDENTIFIER
**Deposited data**		

Short-read human whole genome sequencing data	1000 Genomes Project	HG00096, HG00105, HG00110, HG00130, HG00146, HG02215
1000 Genomes Project RNA-Seq	Taylor et al.^42^	SRA: PRJNA851328
Long-read human whole genome sequencing data	Gustafson et al.^49^	HG00127
rDNA CN (18S Ratio) estimates for UK Biobank participants	Rodriguez-Algarra et al.^14^	Return for UKB application 83271
Non-human apes whole genome sequencing data	Yoo et al.^40^	SRA: PRJNA602326, PRJNA976699–PRJNA976702 and PRJNA986878–PRJNA986879
Human 80S ribosome structure	Holvec et al.^50^	PDB: 8QOI
rDNA IGF estimates for UK Biobank participants	This paper	Return for UKB application 83271

**Chemicals, peptides, and recombinant proteins**		

RPMI 1640	Thermo Fisher Scientific	Cat#11875093
Fetal bovine serum, value	Thermo Fisher Scientific	Cat#A5256701
Penicillin-Streptomycin	Thermo Fisher Scientific	Cat#15140122
Phophate Buffered Saline (PBS)	Thermo Fisher Scientific	Cat#10010023
Tris-HCl, 1M, pH 7.4	Thermo Fisher Scientific	Cat#J60202.K2
MgCl2	Thermo Fisher Scientific	Cat#AM9530G
NaCl	Thermo Fisher Scientific	Cat#AM9759
IGEPAL Ca-630	Sigma-Aldrich	Cat#18896
Sodium Deoxycholate	Sigma-Aldrich	Cat#D6750
RNase free water	Thermo Fisher Scientific	Cat#10977035
Cycloheximide	Thermo Fisher Scientific	Cat#J66004.XF
Dithiothreitol	Sigma-Aldrich	Cat#D0632
RNasin® Ribonuclease Inhibitor	Promega	Cat#N2115
Pierce Protease Inhibitor Mini	Thermo Fisher Scientific	Cat#A32953
UltraPure Sucrose	Thermo Fisher Scientific	Cat#15503022
TRIzol LS	Thermo Fisher Scientific	Cat#10296028
Sodium Acetate	Thermo Fisher Scientific	Cat#R1181

**Critical commercial assays**		

NEBNext® Ultra™ II Directional RNA Library Prep Kit for Illumina®	New England Biolabs	Cat#E7760L

**Experimental models: Cell lines**		

Human lymphoblastoid cell lines	Coriell Institute for Medical Research	HG00096, HG00105, HG00110, HG00130, HG00146, HG02215

**Software and algorithms**		

samtools	Li et al.^51^	https://github.com/samtools/samtools
BBmap	Bushnell^52^	https://sourceforge.net/projects/bbmap/
TrimGalore	Krueger^53^	https://github.com/FelixKrueger/TrimGalore
LoFreq∗	Wilm et al.^32^	https://github.com/CSB5/lofreq/
mason2	Holtgrewe^54^	https://github.com/seqan/seqan/tree/main/apps/mason2
bowtie2	Langmead and Salzberg^55^	https://github.com/BenLangmead/bowtie2
bedtools	Quinlan and Hall^56^	https://github.com/arq5x/bedtools2
minimap2	Li^57^	https://github.com/lh3/minimap2
megalodon	Oxford Nanopore Technologies	https://github.com/nanoporetech/megalodon
PHESANT	Millard et al.^34^	https://github.com/MRCIEU/PHESANT
HairSplitter	Faure et al.^58^	https://github.com/RolandFaure/Hairsplitter
MAFFT	Katoh and Standley^59^	https://mafft.cbrc.jp/alignment/software/
raxml-ng	Stamatakis^60^	https://github.com/amkozlov/raxml-ng
Jalview	Waterhouse et al.^61^	https://www.jalview.org/
R2DT	Sweeney et al.^62^	https://github.com/r2dt-bio/r2dt
ChimeraX	Pettersen et al.^63^	https://github.com/RBVI/ChimeraX
Analysis scripts for this study	This paper	Zenodo: https://doi.org/10.5281/zenodo.18314534

**Other**		

Gradient Master	Biocomp Instruments	Cat#108
Piston Gradient Fractionator	Biocomp Instruments	Cat#152
Triax Flow Cell	Biocomp Instruments	Cat#FC-2-UV/VIS
Fraction Collector	Gilson	Cat#FC-203B
Open-Top Polyclear Centrifuge Tubes	SETON Scientific	Cat#7030

### Experimental model and study participant details

The present study associates phenotypic and genetic data from approximately 500,000 UK Biobank participants recruited between 2006 and 2010, obtained via project application 83271. In addition, the study also relies on publicly-available genetic and transcriptomic data, as well as newly generated data, for 6 GBR participants from the 1000 genomes project.

### Method details

#### Generation and analysis of polysome-seq data

Human lymphoblastoid cell lines (LCLs) were obtained from the NHGRI Sample Repository for Human Genetic Research at the Coriell Institute for Medical Research (New Jersey, USA). Cell lines were seeded at a density of ∼200,000 cells/ml in RPMI 1640 (Gibco Cat. No. 11875093) supplemented with 15% fetal bovine serum (Gibco Cat. No. A5256701) and a 1% penicillin and streptomycin mix (Gibco Cat. No. 15140122). All cell cultures were kept in 37°C incubators under 5% carbon dioxide conditions.

Preparation of LCL cell line lysate was adapted from a public protocol for polysome profiling followed by quantitative PCR.^64^ A minimum of 50 million LCLs were treated with 100μg/ml cycloheximide (Thermo Fisher Scientific Cat. No. J66004.XF) for 15 min at 37°C. Cells were pelleted by centrifugation at 500*g* for 3 min at 4°C, and washed twice with ice-cold PBS (Thermo Fisher Scientific Cat. No. 10010023) supplemented with 100μg/ml cycloheximide. Lysis buffer consisted of 25 mM Tris-HCl (Thermo Fisher Scientific Cat. No. J60202-K2), 5 mM MgCl2 (Thermo Fisher Scientific Cat. No. AM9530G), 100 mM NaCl (Thermo Fisher Scientific Cat. No. AM9759), 1% IGEPAL Ca-630 (Sigma-Aldrich Cat. No. 18896), 1% Sodium deoxycholate (Sigma-Aldrich Cat. No. D6750), in RNase free water (Thermo Fisher Scientific Cat. No. 10977035), adjusted to pH 7.4, and supplemented with 1mM dithiothreitol (Sigma-Aldrich Cat. No. D0632), 40 U/mL RNase inhibitor (Promega Cat. No. N2115) and 1x Pierce Protease Inhibitor Mini tablet (Thermo Fisher Scientific Cat. No. A32953) just before use. Cells were resuspended in 300μL ice-cold lysis buffer, incubated on ice for 30 min with occasional inversion. Lysate was then spun down at 13,000*g* for 10 min at 4°C, supernatant was transferred to a new tube and flash frozen in liquid nitrogen until use.

In order to separate and extract the polysome fraction, sucrose buffer consisted of 25 mM Tris-HCl, 5 mM MgCl2, 100 mM NaCl in RNase free water. 10% or 45% sucrose (Thermo Fisher Scientific Cat. No. 15503022) was dissolved in this buffer, and supplemented with 100 μg/mL cycloheximide, 40 U/mL RNase inhibitor and 1 mM PMSF just before use. 10–45% sucrose gradients were generated using a Gradient Master (Biocomp Instruments) following manufacturer instructions. In brief, the half full point of Open-Top Polyclear Centrifuge Tubes (SETON Scientific Cat. No. 7030) was marked using a Biocomp marker block. 10% sucrose buffer was added to the halfway point, and the 45% sucrose buffer was carefully layered beneath using a blunt needle, to the halfway point. A 2-3mm distance between the buffer and top of the tube was confirmed at this point, adjusting with 10% buffer where necessary. Tubes were then capped with rate zonal caps (BioComp Instruments), and inserted into the Gradient Master tube holder and run using the 10–50% gradient program.

After gradient formation, the level of buffer in each tube was adjusted to be identical in each tube by removal of buffer where necessary. 150μL of sample was layered on top of the sucrose gradient, and centrifuged at 40,000 rpm (273,620g) for 1.5 h at 4°C using an SW41 Ti rotor (Beckman Coulter Cat No. 331362) in a Sorvall WX + Ultracentrifuge (Thermo Fisher Scientific). Gradients were then profiled using the combined Piston Gradient Fractionator and Triax Flow Cell (BioComp Instrument), and fractions were collected using an FC-203B Fraction Collector (Gilson). Based on the 254 nm absorbance recorded by the Gradient Fractionator, polysome fractions were taken from mRNAs associated with 3 or more ribosomes. Five fractions were taken from this point, pooled, and TRIzol LS Reagent (Thermo Fisher Scientific Cat. No. 10296028) was added prior to storage at −20°C.

RNA was isolated from lysate and polysome fractions using TRIzol LS Reagent following manufacturer’s instructions. Samples were further cleaned up using sodium acetate (Thermo Fisher Scientific Cat. No. R1181) precipitation.^65^

Paired-end short read sequencing libraries were generated using NEBNext Ultra II Directional RNA Library Prep Kit for Illumina, NEB, USA. Sequencing was performed on an Illumina NovaSeq X Plus Series by Novogene, Cambridge, UK. A list of samples and corresponding QC metrics is included in Table S4.

### Quantification and statistical analysis

#### General statistical analysis

Unless otherwise specified, all statistical analyses were conducted using in-house R scripts (version 4.5.1) relying on the data manipulation and visualisation packages from the tidyverse version 2.0.0.^66^ Differences of means were tested using Wilcoxon signed-rank tests from R’s wilcox.test() function, including the paired = TRUE option when necessary. Correlation coefficients and corresponding *p* values were derived using Pearson’s method from the cor.test() function. Adjustment for multiple tests was conducted using the p.adjust function from R, with method = ‘fdr’. When “global FDR” is indicated, the adjustment was conducted including all comparisons (e.g., for all combinations of variant and phenotype). Alternatively, when “variant-level FDR” is mentioned, the adjustment was conducted separately for each variant, so with one entry per phenotype tested.

#### Variables of interest and covariates

Phenotypic data and sequencing metadata for UKB participants included in the association models was obtained as previously reported.^14^ In particular, Sex was obtained from UKB field ID 31, with “0” representing females and “1” males. Age at recruitment was obtained from field ID 21003, with Age Squared derived as the value multiplied by itself. Sequencing Center information was obtained from field ID 32051. Assessment Center recorded in field ID 54, Adjusted Telomere Length in field ID 22191, and the 10 first Genetic Principal Components in field ID 22009, were also included as covariates in all analyses. Participants with a value of “1001” in field ID 21000 and “1” in field ID 22006 were identified as “White British”.

#### Identification of relatives and unrelated UKB participants

Using the genetic relatedness information that the UKB provides for 107,076 pairs of participants, with 147,612 individual participants represented, we identify monozygotic twin pairs and construct a set of “fully unrelated” participants. In particular, entries in the relatedness table with kinship >0.4 were considered monozygotic twins. From these, comparison between rDNA variant frequencies in relatives were limited to pairs where both individuals were identified as “White British” and their sequencing data was generated by the same center within the same release. The entries of the relatedness table were also employed to create a set of 297,010 unrelated individuals by keeping one participant from each identified “family”, as previously described.^14^

#### Access to sequencing data and computations in the UKB RAP

rDNA intragenomic variant frequency estimates were calculated for all WGS samples available for both first and second UKB releases, similarly to how proxy rDNA CN estimates had been calculated previously.^14^ Alignment files in cram format were accessed through the UK Biobank Research Analysis Platform (UKB RAP). Since the second sequencing release was made available, these are stored in subfolders the/Bulk/GATK and GraphTyper WGS/Whole genome GATK CRAM files and indices [500k release]/path. To enable parallelisation of the computation, the paths to the cram files were split into batches and saved in separate files with up to 10,000 participants each. Reads identified as mapping to rDNA-analogue regions of the Hg38 assembly (see below) were extracted using samtools.^51^. This was launched using a pair of scripts (one local and another remote) via the UKB RAP application called swiss-army-knife (SAK) version 4.5.0. The two scripts communicated through the dxpy python library, installed locally on a virtual environment (via pip install dxpy). Multiple SAK instances can be executed including in the submission script the options -iin="${project}${script_path}" and -icmd="bash '${script}' '$i'", as previously described.^14^ The remote script then calls samtools to extract the reads in the region of interest as a bam file for each cram file assigned to its instance (see “Identification of rDNA-analogue regions” below). The paths to each alignment file were prepended with/mnt/project/, which enables their processing without needing to previously copy them. The resulting filtered alignment files were then further processed to obtain the variant frequency estimates as detailed below.

#### Processing of short-read sequencing data

Retrieved short-read bam alignment files were converted back to fastq format using samtools, and then processed using the repair.sh script from BBMap version 38.95^52^ including the -Xmx20g options to remove singleton reads. The output was then processed using trimgalore version 0.6.5^53^ in --paired mode and aligned to the appropriate reference for each analysis using bowtie2. In particular, alignments to the rDNA unit were conducted using a GenBank: KY962518.1 rDNA consensus reference with the last 2120 bases removed and then prepended at the start of the sequence. This same “looped” reference was incorporated into a masked Hg38 reference assembly, as previously described.^14^^,^^25^ George et al.^26^ employed a similar procedure to construct the alternative WG + rDNA reference sequences in Figure S1. The main difference between their Hg38+rDNA and ours is the stringency of the similarity with rDNA sequence required, with theirs removing around 880k of sequence from Hg38 whereas ours masks around 490k in the same regions where the rDNA reads align in Figure S6A.

Intragenomic variant frequencies throughout the rDNA transcriptional unit were obtained from the bowtie2 alignments using LoFreq version 2.1.5.^32^ Similar to the procedure described in our prior work,^25^ this is conducted in two steps, first obtaining a putative list of “high confidence” variants and then retrieving “exhaustive” frequency estimates. In particular, rDNA alignments were quality-annotated using lofreq alnqual and then processed with lofreq call --call-indels --use-orphan. The resulting calls were then fed to lofreq filter -v 2000 -a 0.1 to obtain a set of “high confidence” variants, where -v indicates a mininum read depth and -a a minimum intra-genomic frequency for a variant to be reported on any given sample. Additionally, lofreq call was also executed including the -a 1 -b 1 -B --no-default-filter options, which provides an “exhaustive” list of all observed variant frequencies in a sample. In this case, -a indicates a minimum *p* value threshold and -b a “Bonferroni factor” to adjust the obtained *p* values, so providing 1 in both removes any significance filtering. Moreover, -B disables the use of base alignment quality. The goal of these parameters is to retrieve as many variation estimates as possible, even on noisy positions. The reliability of variant calls will then be determined according to their presence on other samples. In particular, variants present in the “high confidence” output of at least one MZ twin pair were then retrieved from the “exhaustive” output for each sample. Frequencies of 0 were then imputed for all “high confidence” variants absent from the exhaustive mode.

#### Location of rDNA-analogue regions

Two complementary procedures were employed to identify the regions of the Hg38 assembly used in the UKB alignments where reads originating from the rDNA clusters map. First, we generated 1,000,000 simulated Illumina paired-end reads of 150 bp each from the GenBank: KY962518.1 rDNA reference using mason2 version 2.0.9.^54^ These were then aligned to the unmodified Hg38 assembly present in the UKB (file GRCh38_full_analysis_set_plus_decoy_hla.fa, corresponding to the assembly previously employed in the 1000 Genomes Project) using bowtie2 version 2.4.5.^55^ Read coverage across all Hg38 contigs was then calculated using samtools depth from samtools version 1.19.0, whose output was then employed to determine which regions accumulate more alignments. Second, we converted the UKB alignments to Hg38 for the same-centre MZ twin pair samples included in the second WGS release to bed format using the bamtobed tool from bedtools version 2.28.0.^56^ We then realigned those same samples exclusively to the GenBank: KY962518.1 rDNA reference using bowtie2. These new alignments were also converted to bed format. Comparing the mapping locations on a per-read basis from the two generated bed files thus provided the regions where rDNA-mapping reads accumulate in Hg38. The two procedures provided a consistent set of coordinates we then employ to extract reads mapping to rDNA-analogue regions from the UKB alignments (Table S5).

#### Short-read sequencing data from the 1000 Genomes Project

We retrieved paired-end 1000 Genomes Project (1kGP) WGS fastq files from the sequence_read folders within the gridftp/1000g/ftp/phase3/data/path for the GBR individuals available through the EMBL-EBI public Globus endpoint. Ignoring those generated using ABI SOLiD technologies and those with insufficient read depth, this represents 84 different GBR participants. The downloaded files were then merged according to their sample and strand of origin.

RNA-Seq data for 28 different 1kGP GBR participants was obtained from the Sequence Read Archive (accession SRA: PRJNA851328).^42^ Of these, 6 were also present in our own generated polysome-seq data and were used in further analyses.

Aside from the UKB WGS data, the short-read processing procedure detailed above was employed for both WGS and RNA-Seq data from the 1kGP. Alignments generated from an RNA-specific aligner, such as STAR, cannot be processed with LoFreq, and alternative software tools require specific phased transcripts to quantify. The combination of bowtie2 and LoFreq, on the other hand, provides the intended output even if the source of the data is not DNA since no splicing variants are expected in the 47S rDNA loci.

#### Oxford Nanopore sequencing data from the 1000 Genomes Project

ONT data for participant HG00127 from the 1kGP was retrieved on the 12th of March, 2024 in fast5 format from the repository at https://s3.amazonaws.com/1000g-ont/index.html^49^ At the time of writing, however, only files in pod5 format are present in the repository, having been converted to the most recent ONT file format. For backwards compatibility, the current file format can be reverted to fast5 format using the pod5 convert to_fast5 tool provided by ONT.^67^ The fastq files included in the fast5 were then aligned to the Hg38+rDNA reference using minimap2 version 2.26.^57^^,^^68^. Reads mapping to the 28S rDNA were identified from these alignments using samtools.

Similar to the procedure reported before,^25^ variant frequencies in ONT data were estimated from the output table generated by megalodon version 2.2.9^69^ with the --outputs per_read_variants option. Variants within the 28S identified in the UKB MZ twins and detected in the WGS data for HG00127 were provided alongside the list of 28S-mapping reads generated above. Only per-read variant calls reported with probability exceeding 0.9 were retained.

#### Phenotypic association models

Phenotypic association screens were conducted using PHESANT^34^ on the set of 297,010 unrelated WB UKB participants for both rDNA sequence and total CN variation (in the form of the 18S Ratios previously calculated^14^). The analysis was restricted to a more stringent set of variables than previously reported^14^ (Table S6). The selected fields were then processed as PHESANT requires, prepending an “x” and replacing dashes with underscores in their names.

The phenomeScan.r script from PHESANT was executed on R version 4.2.2 for each variable of interest. This fits regression models tailored to the type of phenotype under consideration, such as linear models for continuous values and binomial models for binary traits. Sex, age, age squared, sequencing center and release, assessment center, the first 10 genetic principal components, and adjusted telomere length were used as covariates, and the --genetic="FALSE" option was included, since the principal components were explicitly provided in the covariate table. The script was run for each analysis in 100 parts, which were then combined using the mainCombineResults.r script. Overall, PHESANT generated output for 429 distinct phenotypes for the CN associations and 419 across the variant frequency associations, although the number varies depending on the particular variant.

Linear regression models using R’s lm() function were fit to compare the effect size estimates in Figures S11 and S12, and the differences in Figures S13 and S15. The same covariates described above were included in these models, plus the rDNA CN (in the form of 18S Ratios) as additional covariate when appropriate (such as in Figure 4C). In particular, the regression models for each variant and trait pair adhere to the following formulation:

$$Trait\;∼\;Variable+Sex+Age+{Age}^{2}+AssessmentCentre+SequencingCentre+SequencingRelease+PC\left\{1-10\right\}+AdjTelLength\;\left(+CN\right)$$

where “Variable” is either the intragenomic variant frequency or the allele-specific copy number, PC{1–10} are all 10 first genetic principal components, “AdjTelLength” is the adjusted telomere length estimates and the parentheses indicate that the rDNA CN was only included in selected analyses.

#### Construction of ES15L haplotypes

An in-house python script called var_extract was developed to retrieve the alleles present at a given set of positions for each read mapping in the region. This script was constructed similarly to the previously released allele-specific methylation tool blink,^25^ but extending its functionality to enable the identification of indels aside from SNVs. To this end, var_extract relies on the pysam library to traverse each mapped position in a read and parse the base (or sequence of bases) present at the specific reference positions of interest. The output table can then be processed to determine colocalisation between variants in single reads and thus derive the most prevalent “haplotypes” in the region. The similarity between the per-variant frequencies derived from these per-read extractions and those LoFreq estimates supports the validity of the extraction procedure (Figure S24).

#### Analysis of non-human ape sequences

We located the region equivalent to the human ES15L through the flanking conserved sequences (CGCCGGCAGTCAGTCG and GGGCGCGGGCCCGGGT) in the 28S of the recently published non-human ape genomes.^40^ To identify common variants, we extracted all HiFi sequencing reads originating from this region and generated haplotypes using hairsplitter.^58^ The output was filtered to retain only the haplotypes with a minimum read support (5% of the total reads assigned to haplotypes by hairsplitter). The reference haplotype for each species was also included (labeled REF in Figure S17; chimpanzee [GenBank: PQ407870.1]; bonobo [GenBank: PQ407869.1]; western gorilla [GenBank: PQ407868.1]; Bornean orangutan [GenBank: PQ407872.1]; Sumatran orangutan [GenBank: PQ407871.1]; and siamang [GenBank: PQ407873.1]). For both bonobo and chimpanzee, the reference haplotypes had less than 5% read support but were retained anyway.

The generated haplotype sequences between (and including) the two conserved fragments above were joined into a fasta file alongside the corresponding sequences for the 10 human haplotypes in Figure 5B. The FASTA file was then fed to the Multiple Alignment using Fast Fourier Transform (MAFFT) EBI tool (https://www.ebi.ac.uk/jdispatcher/msa/mafft), and then to the command line tool mafft-linsi version 7.526^59^ with parameters --thread 8 --op 0.5 --ep 0.0 --lexp -1.0. raxml-ng version 1.2.0^60^ with --model HKY+G was then employed to generate the corresponding phylogenetic tree. This was then processed in R using the ggtree package,^70^ re-rooting it to the siamang’s reference haplotype using the root() function from the ape package.^71^ The multi-alignment result displayed in Figure 5C was visualised using JalView version 2.11.5.0.^61^

#### rRNA structural models

To visualise the potential impact of ES15L variants on its structure, 2D RNA maps were first generated with the RNA 2D Templates (R2DT)^62^ tool and edited using RNAcanvas. The human 80S ribosome structure was visualised with UCSF ChimeraX version 1.8,^63^ using the PDB accession 8QOI.^50^

## Supplemental information

Document S1. Figures S1–S24

Table S1. rDNA variants selected for analysis, related to Figure 2

Table S2. Trait associations with rDNA intragenomic variant frequencies at FDR < 0.01, related to Figure 3

Table S3. Trait associations with rDNA copy numbers at FDR < 0.01, related to Figure 4

Table S4. Quality control metrics for input RNA and polysome-seq data, related to STAR Methods

Table S5. Locations in Hg38 analog to the human rDNA reference, related to STAR Methods

Table S6. List of UKB traits considered in association tests, related to STAR Methods

Document S2. Article plus supplemental information
