# Supplementary material for: Germline sequence variation within the ribosomal DNA is associated with human complex traits
Source: Cell Genom. 2026 Apr 10;6(6):101213. doi: 10.1016/j.xgen.2026.101213 (PMC13261666; doi:10.1016/j.xgen.2026.101213)
Supplement: Document S1. Figures S1–S24 [file mmc1.pdf]

Cell Genomics, Volume *6*

Supplemental information

Germline sequence variation

within the ribosomal DNA

is associated with human complex traits

Francisco Rodriguez-Algarra, Elliott Whittaker, Maia Cooper, Sergey Koren, Maria R. Conte, Adam M. Phillippy, Faraz K. Mardakheh, David M. Evans, and Vardhman K. Rakyan

Supplemental Figures

a)

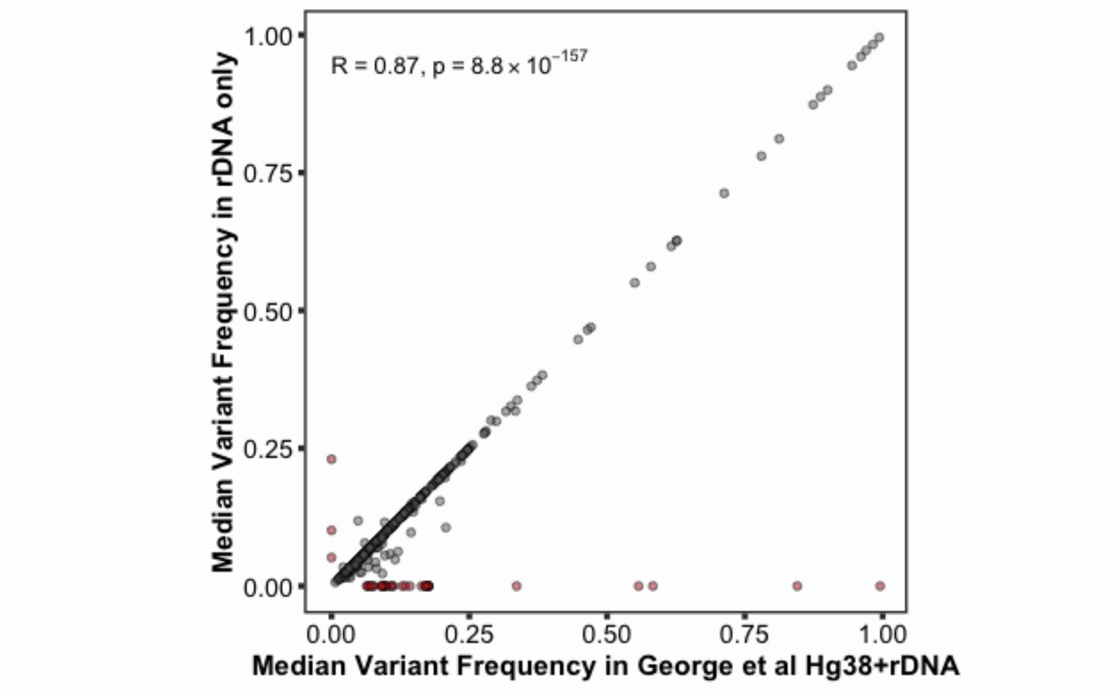

b)

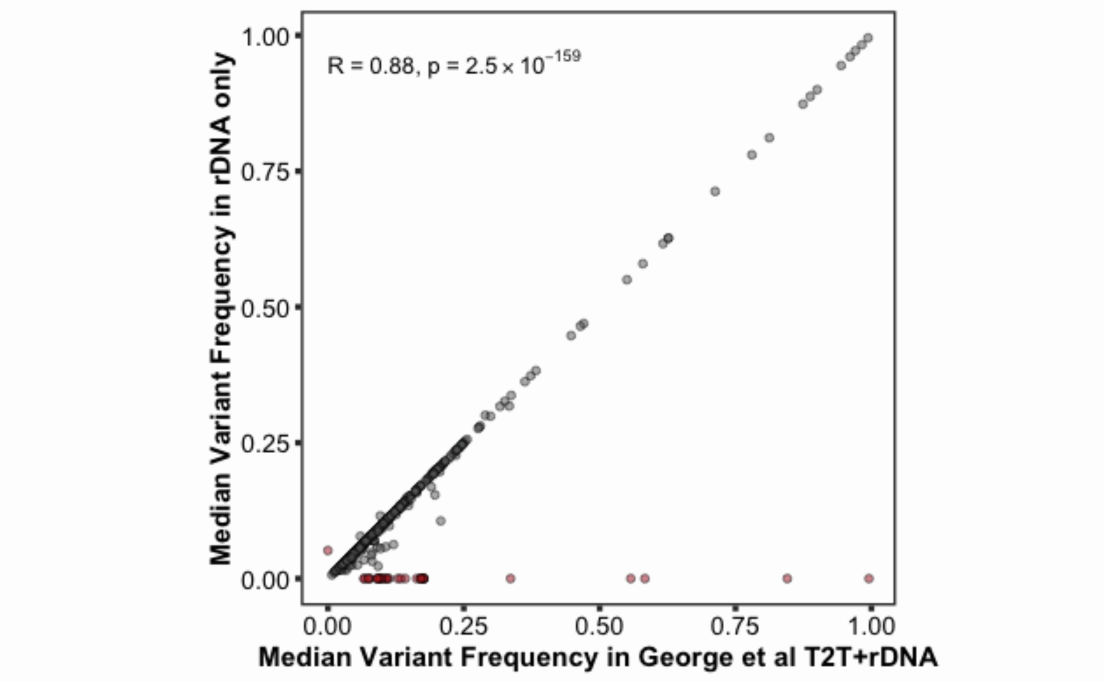

Figure S1: Validation of realignment references, related to Figure 2 and STAR Methods. Comparison between the median frequency for each identified variant in the rDNA transcriptional unit (Pearson's R, N = 535 variants) estimated from realignments of MZ twin samples from the second UKB sequencing release to two full WG+rDNA consensus assemblies generated by George et al[26], and a looped KY962518.1 rDNA reference. Red dots indicate variants that were not detected at any frequency in only one of the approaches.

a)

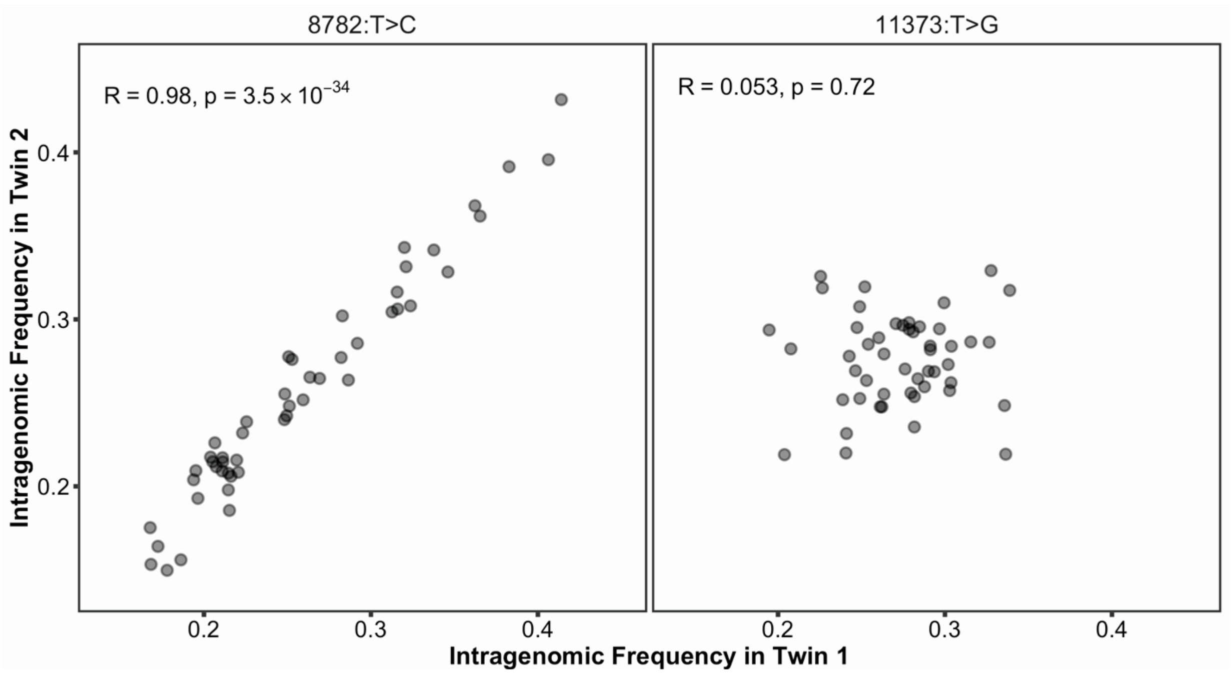

b)

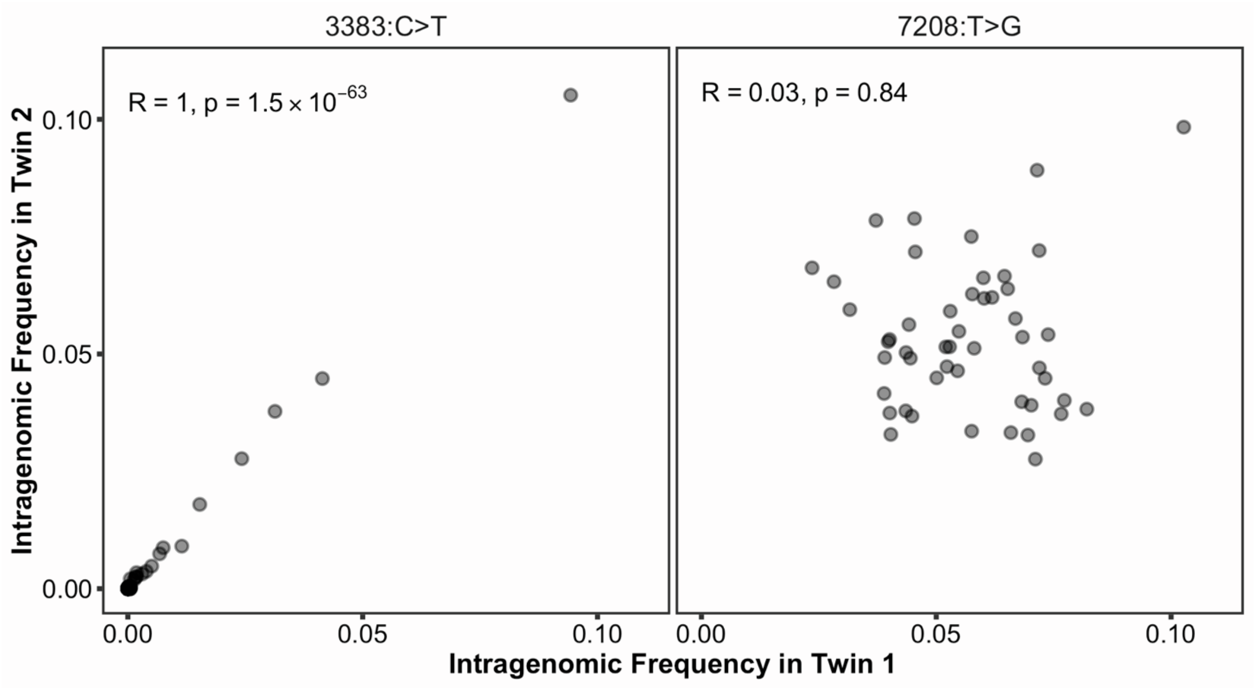

Figure S2: Additional intra-twin-pair IGF comparisons, related to Figure 2. Comparison of concordant (left) and discordant (right) variant positions according to intra-twin-pair IGF correlations (Pearson's R, N = 49 twin pairs) for variant positions with mean IGF **(a)** approximately 0.3 and **(b)** below 0.1. Interpretation as in **Fig. 2B**.

a)

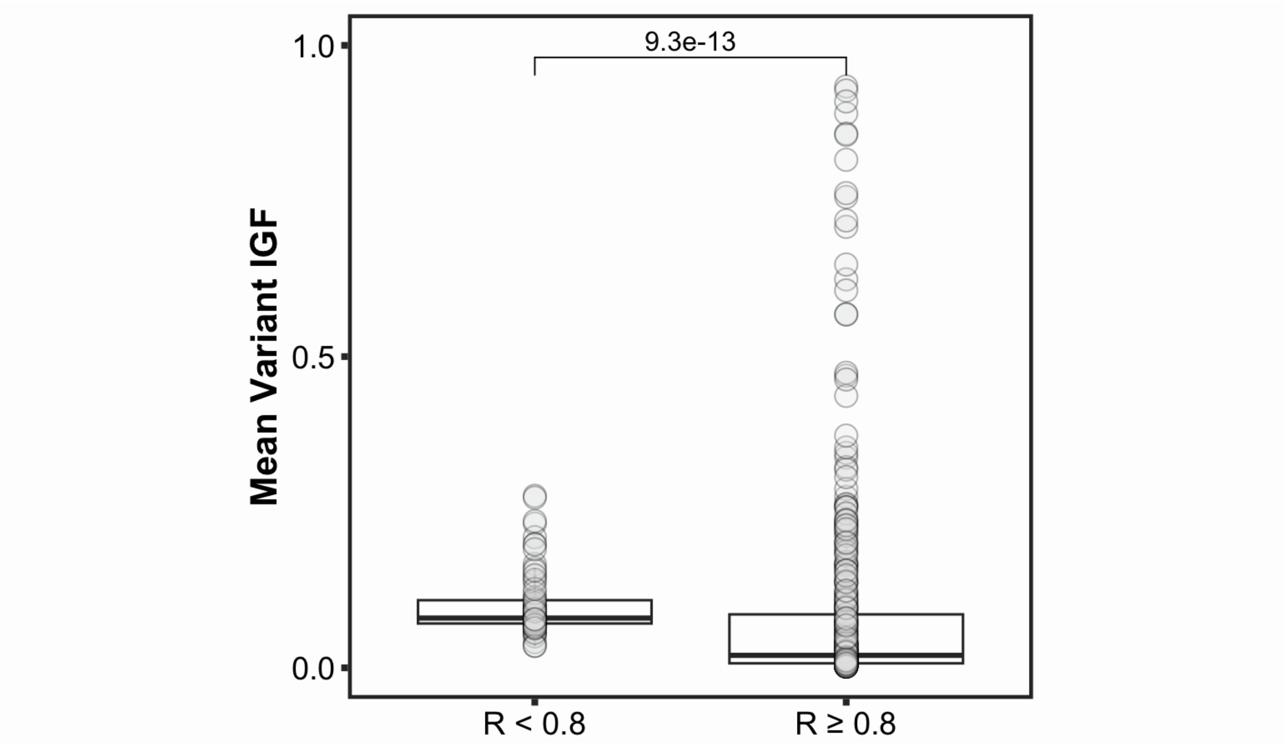

b)

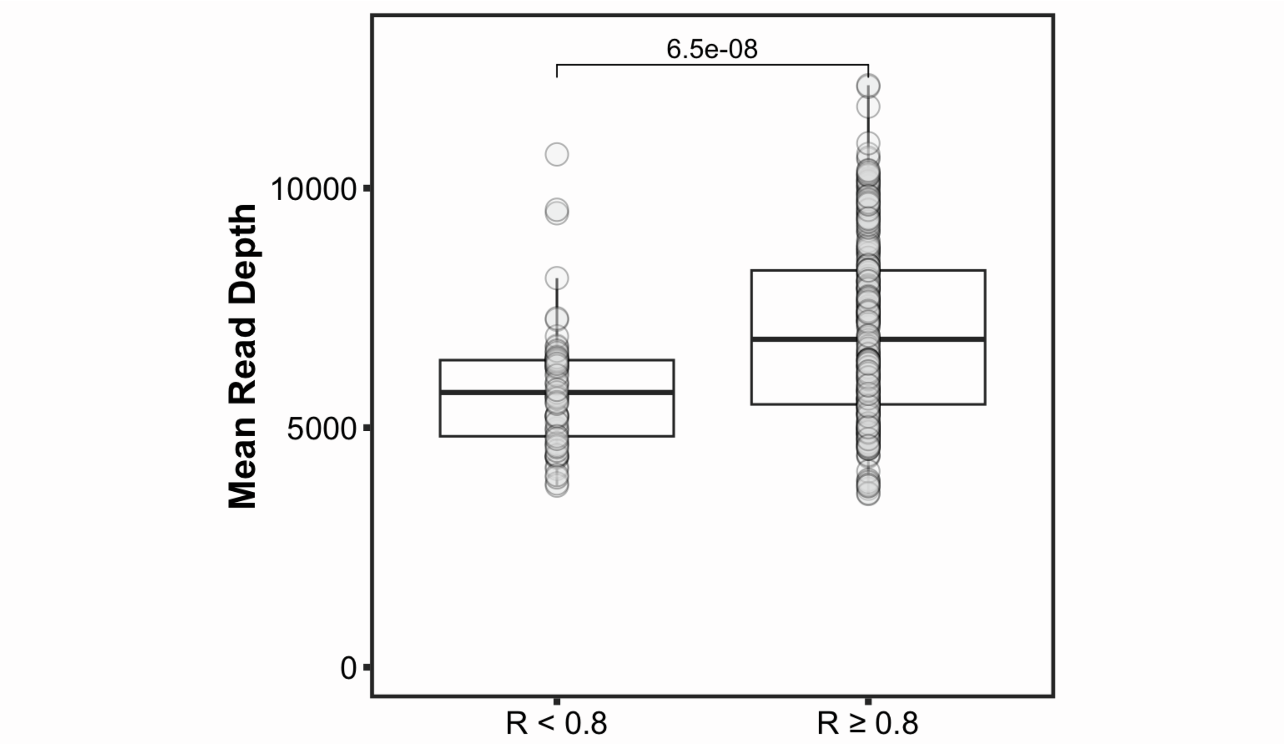

Figure S3: Comparison of IGF and read depth for concordant and discordant variants; related to Figure 2. For variants above and below the 0.8 intra-twin-pair correlation threshold in **Fig. 2C**, comparison of their mean **(a)** estimated intra-genomic frequency and **(b)** read depth. p-value for Wilcoxon rank sum test for the difference of means indicated in each panel ($N_{R<0.8}$ = 77, $N_{R≥0.8}$ = 380).

a)

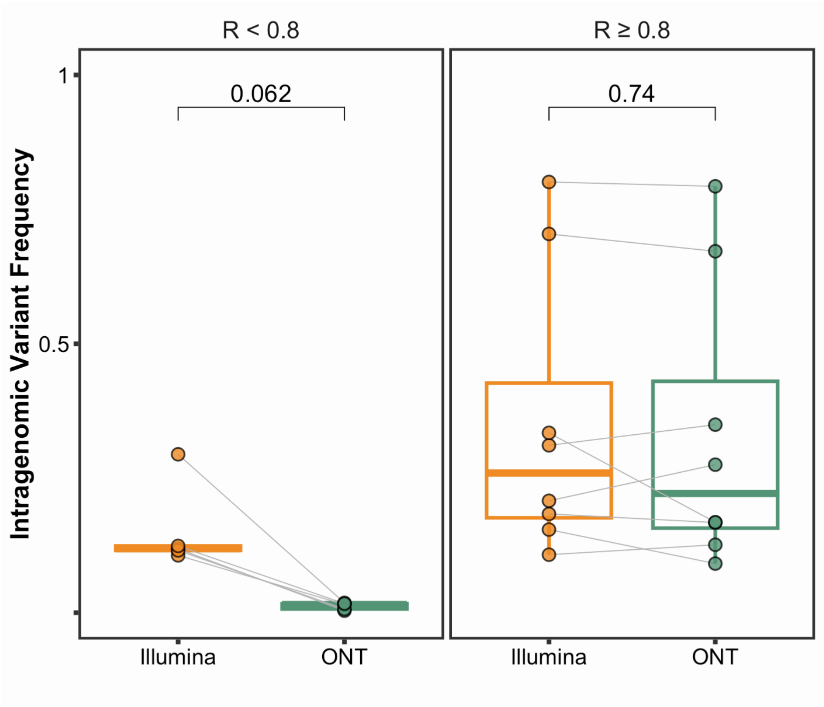

b)

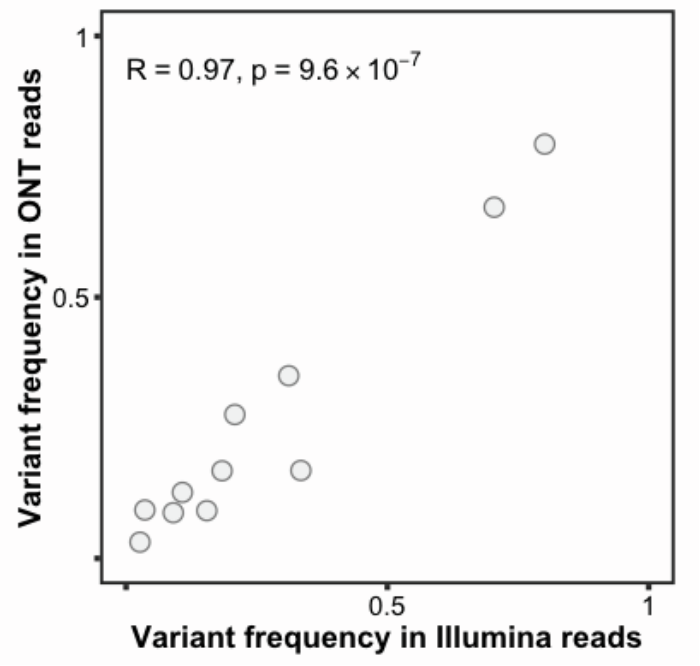

Figure S4: Comparison between Illumina and ONT IGF estimates, related to Figure 2. (a) Comparison between the 28S variant frequencies detected from Illumina short-read and Oxford Nanopore (ONT) long-read for HG00127, split by the intra-twin-pair correlation of each variant obtained on UKB MZ twins, for variants detected in short reads at IGF > 0.1 (subset from **Fig. 2D**). p-value for paired Wilcoxon rank test for the difference in means indicated in each panel ($N_{R<0.8} = 5$, $N_{R\geq0.8} = 8$). **(b)** For 28S variants with UKB intra-twin-pair correlation greater or equal than 0.8, correlation (Pearson's R, N = 11) between the intragenomic variant frequencies estimated for HG00127 in Illumina short-read and ONT long-read data.

a)

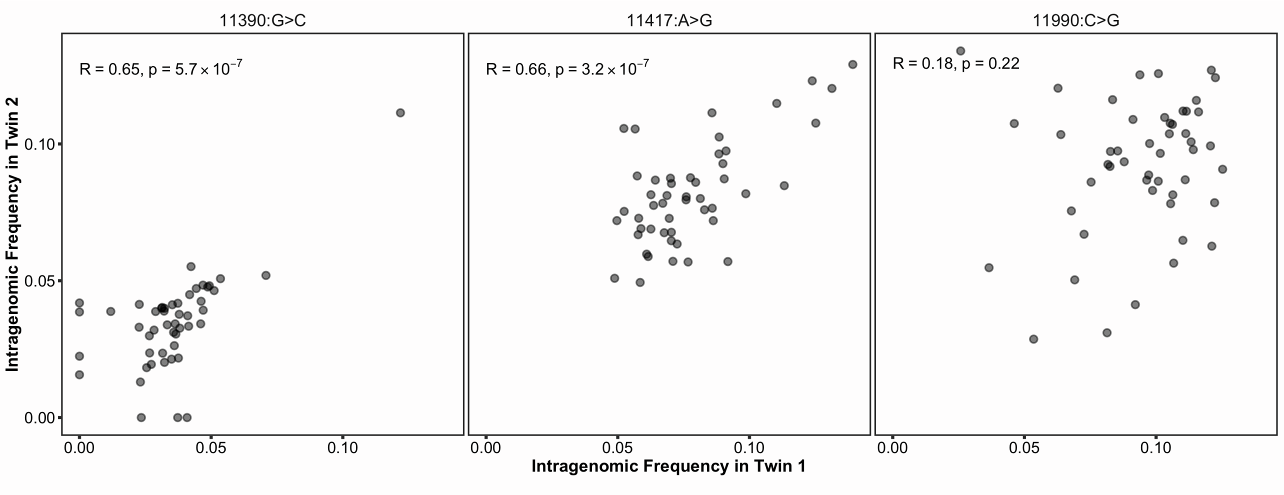

b)

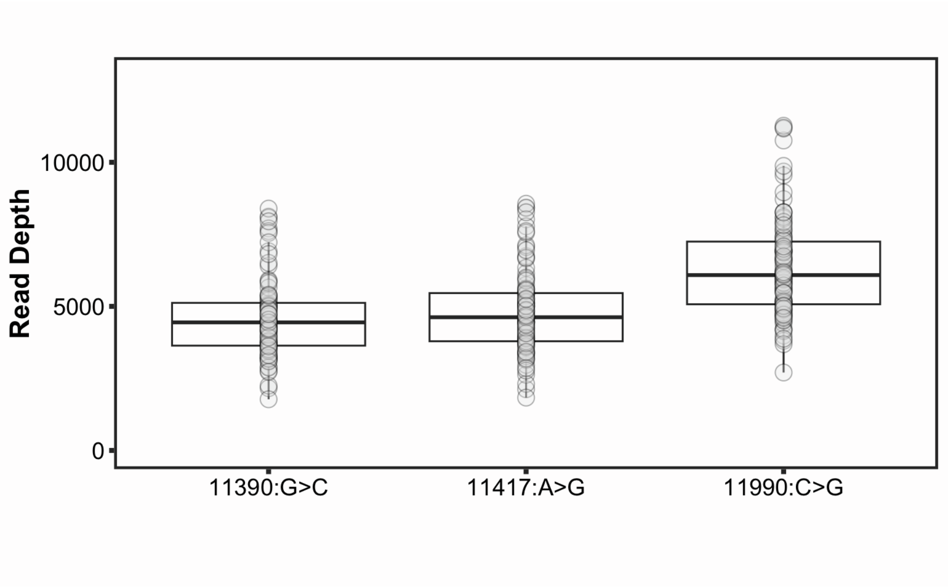

Figure S5: Characteristics of non-T>G/A>C discordant variants, related to Figure 2. (a) Intra-twin pair comparisons (Pearson's R, N = 49 twin pairs) of IGF estimates and **(b)** read depth distributions for the three variants with R < 0.8 in **Fig. 2C** that are neither T>G nor A>C.

a)

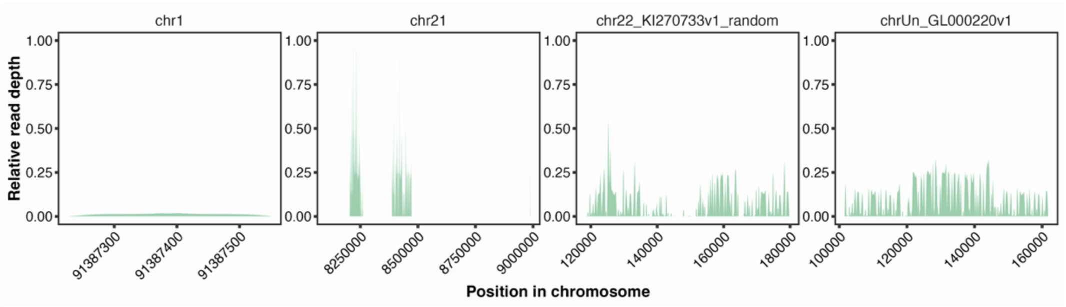

b)

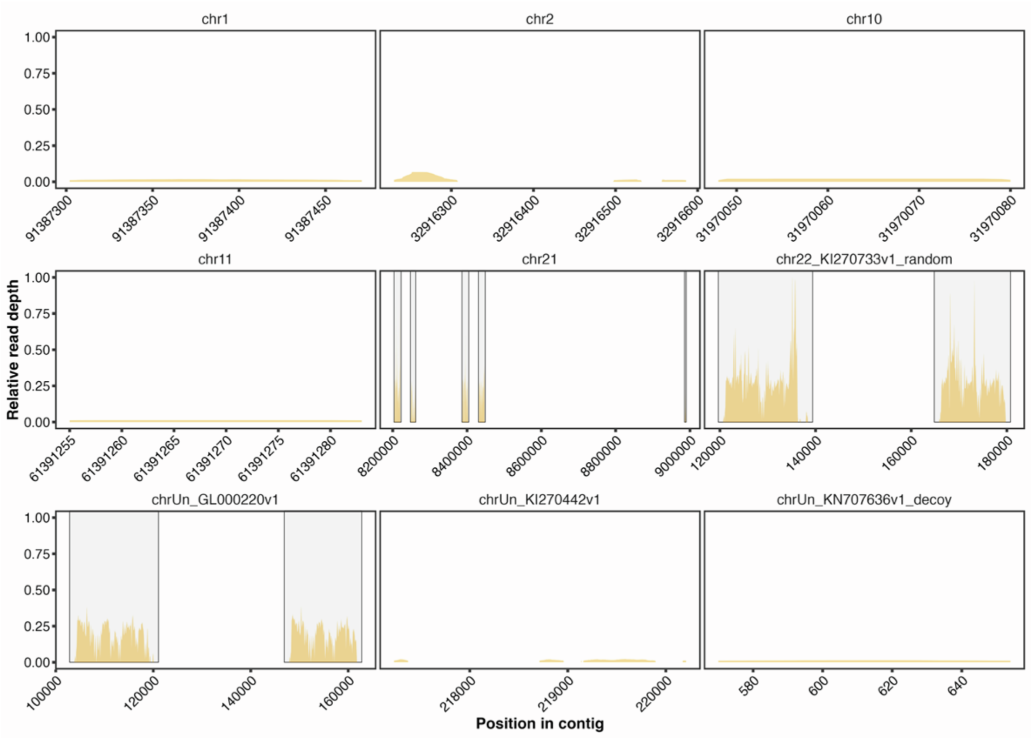

Figure S6: Identification of rDNA analogues in Hg38, related to Figure 2 and STAR Methods. **(a)** For Illumina reads simulated from the entire KY962518.1 rDNA reference and aligned to the Hg38 reference assembly, mapping prevalence across Hg38 contigs (i.e., mapping depth divided by the maximum observed read depth), showing where most simulated rDNA reads map when aligned to the Hg38 assembly. **(b)** Distribution of the origin loci across Hg38 contigs of reads from the UKB prealigned files that map to the rDNA transcriptional unit (the region of interest for the current study) after realignment to a Hg38+rDNA reference. Regions within grey boxes contain >98% of the reads. In addition, all these boxed regions overlap with regions identified using simulated reads in **a**, and are thus considered Hg38 analogues to the rDNA transcriptional unit. All further analysis relying on UKB data within the present study thus solely retain reads mapping to these boxed regions in the prealigned files to estimate rDNA variation. .

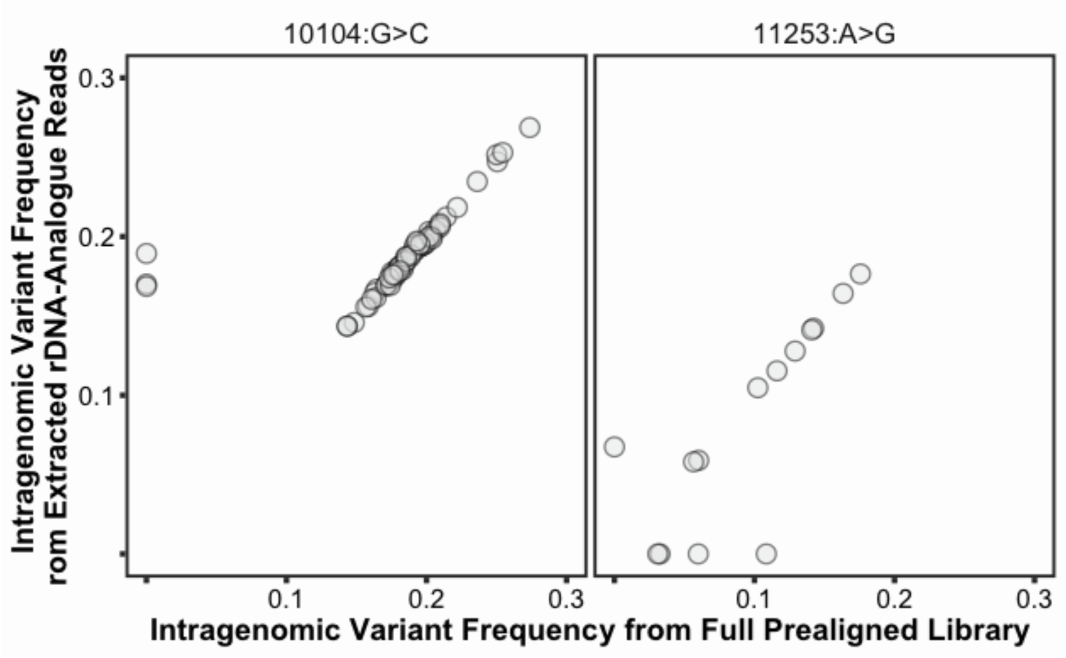

Figure S7: Effect of rDNA analogue extraction in two discarded variants, related to Figure 2. Comparison between the intragenomic frequencies in UKB MZ twin pairs obtained from full library realignments and prior extraction of reads from rDNA analogues, for two variants with R < 0.8 in such comparison.

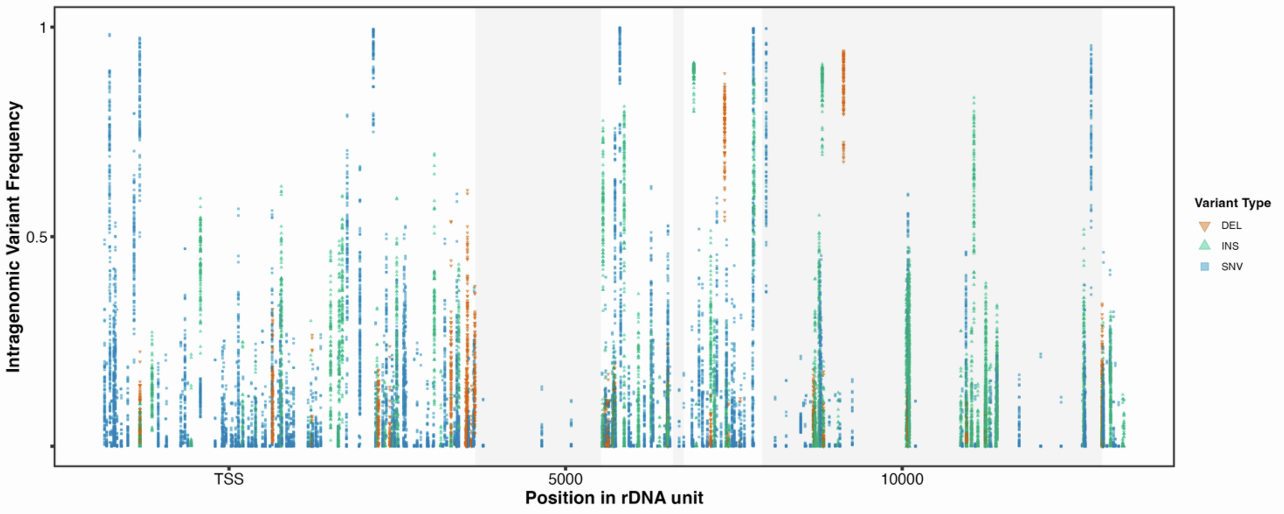

Figure S8: Twin IGFs for variants selected for further analysis, related to Figure 2. Distribution of intragenomic variant frequencies in 47 MZ twin pairs across the rDNA transcriptional unit for variants selected for further analysis. The shadowed regions mark (from left to right) the 18S, 5.8, and 28S.

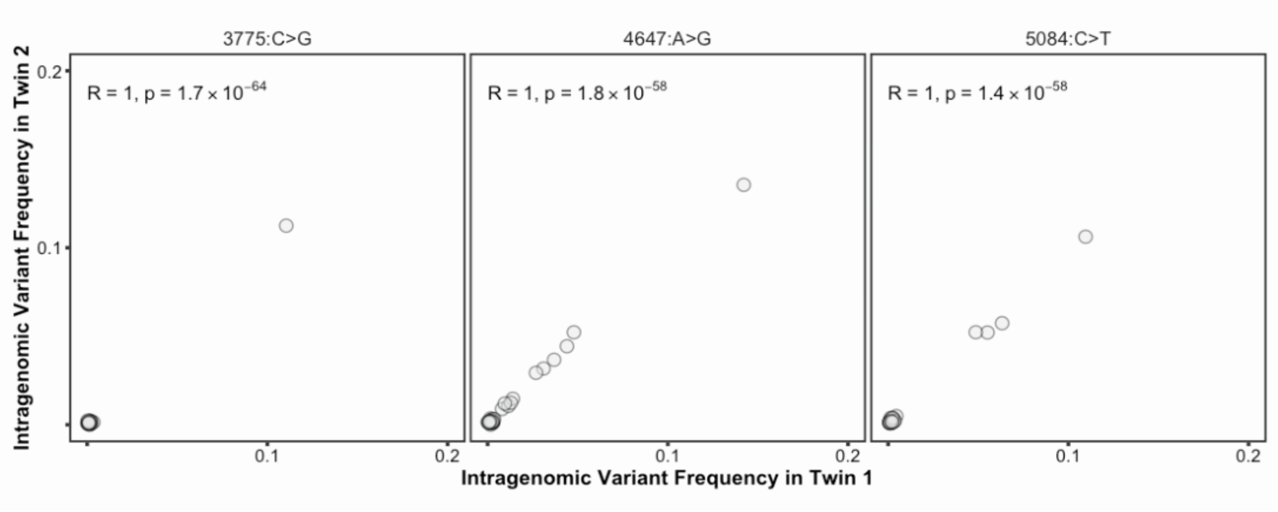

Figure S9: IGF in twin. pairs for 18S variant positions, related to Figure 2. Comparison of estimated intragenomic variant frequencies in MZ twin pairs (Pearson's R, N = 49 twin pairs) for selected variants from within the 18S.

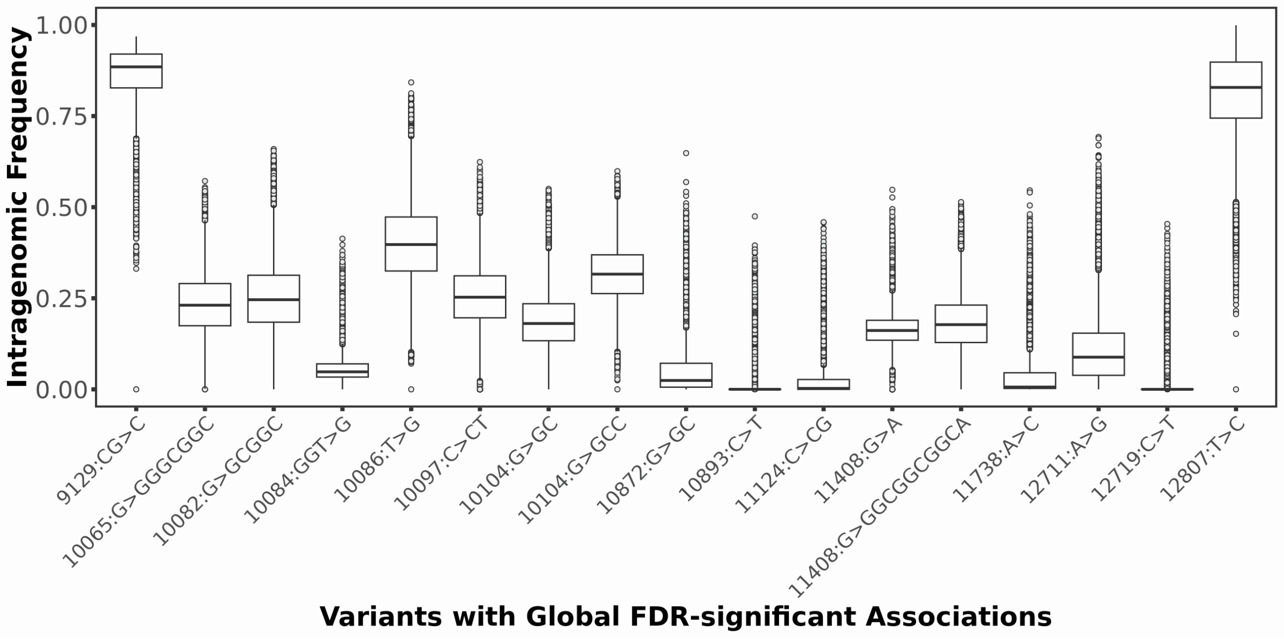

Figure S10: IGF distributions for variants with global FDR-significant trait associations, related to Figure 3. Intragenomic variant frequency distributions across white British UKB participants of rDNA variants reaching global FDR-significant trait associations in **Fig. 3A**.

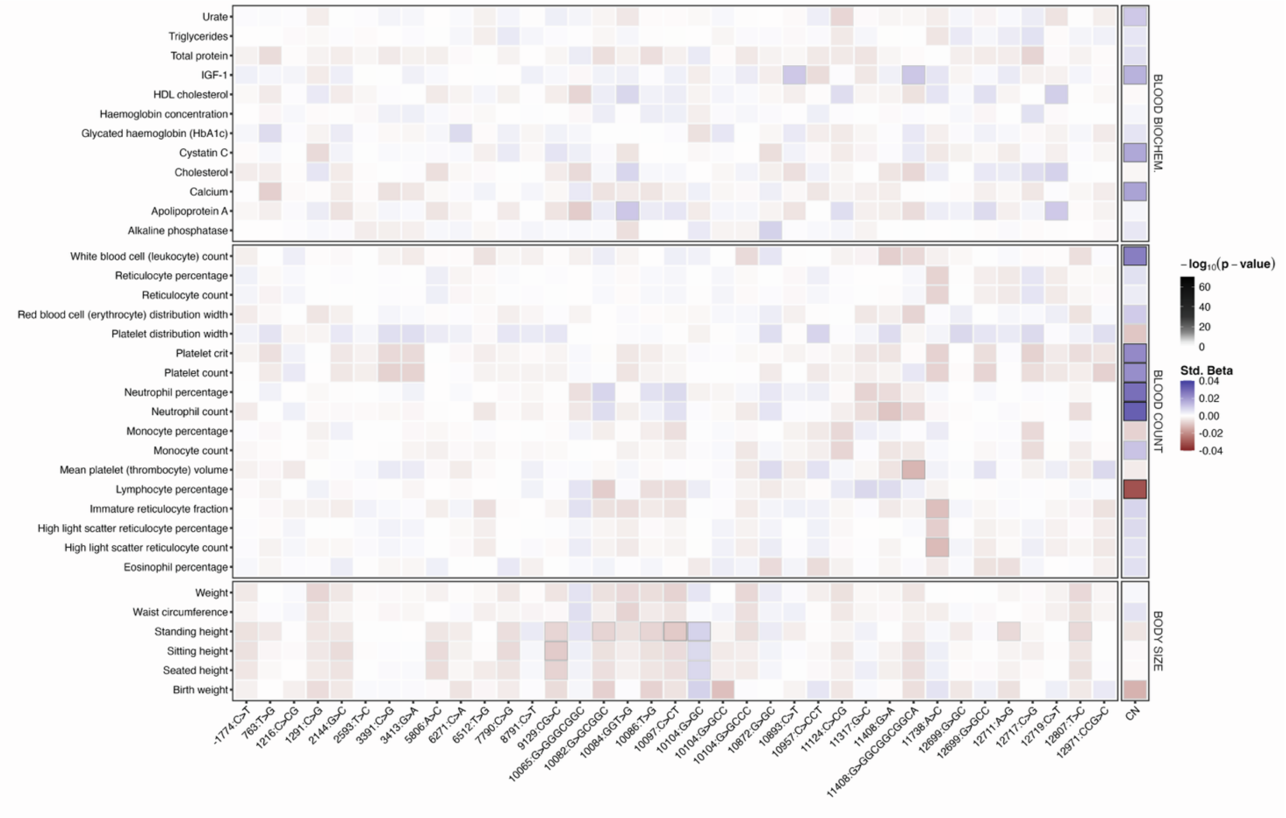

Figure S11: Effect sizes of IGF in a selection of variants and traits, related to Figure 4. Heatmap of effect sizes obtained from regression models of variant frequency for combinations of variants and phenotypes with variant-level associations included in **Fig. 3B** (left) plus the corresponding effect sizes obtained in total rDNA CN associations for the same phenotypes (right).

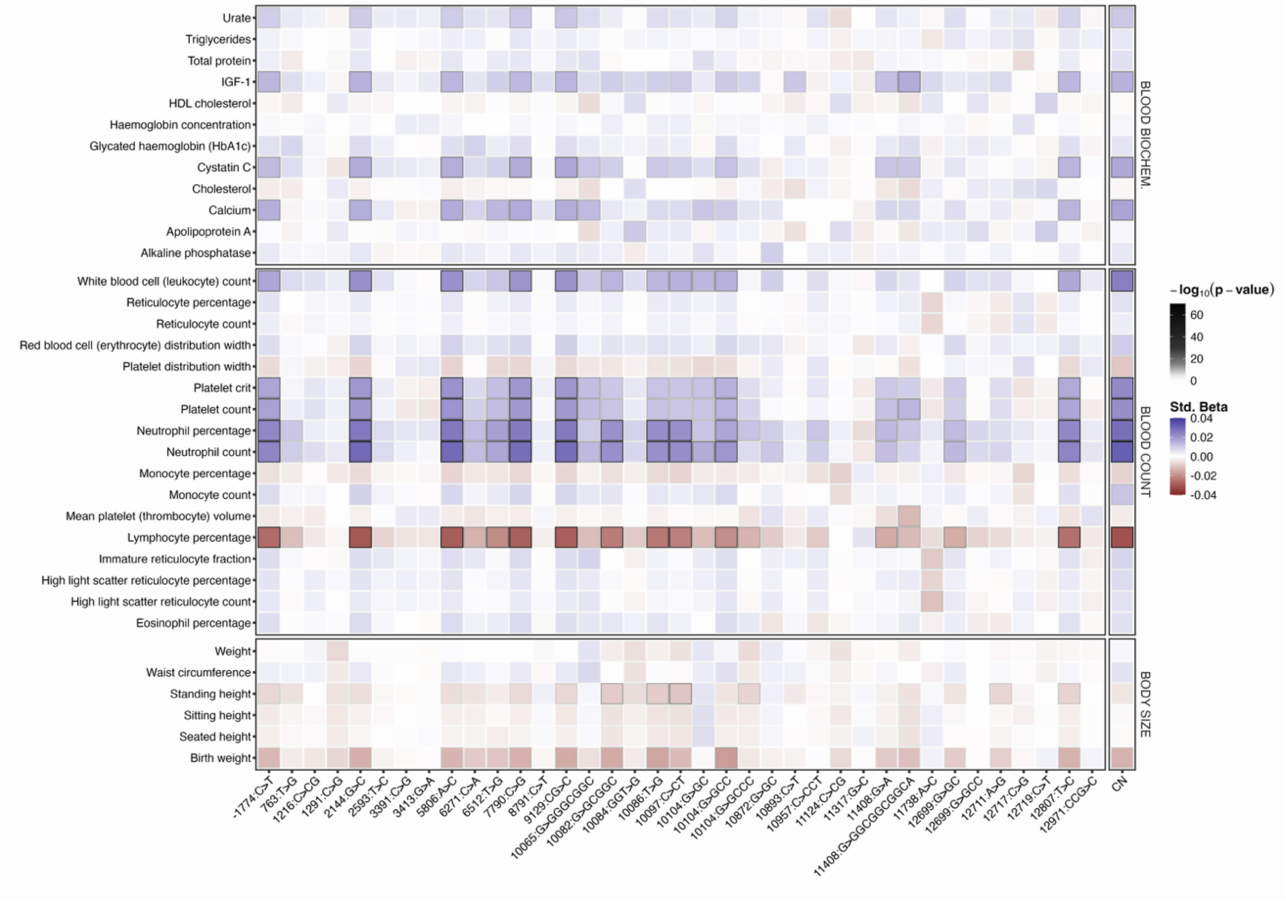

Figure S12: Effect sizes of allele-specific CN in a selection of variants and traits, related to Figure 4. Heatmap of effect sizes obtained from regression models of allele-specific copy number for combinations of variants and phenotypes with variant-level associations included in **Fig. 3B** (left) plus the corresponding effect sizes obtained in total rDNA CN associations for the same phenotypes (right).

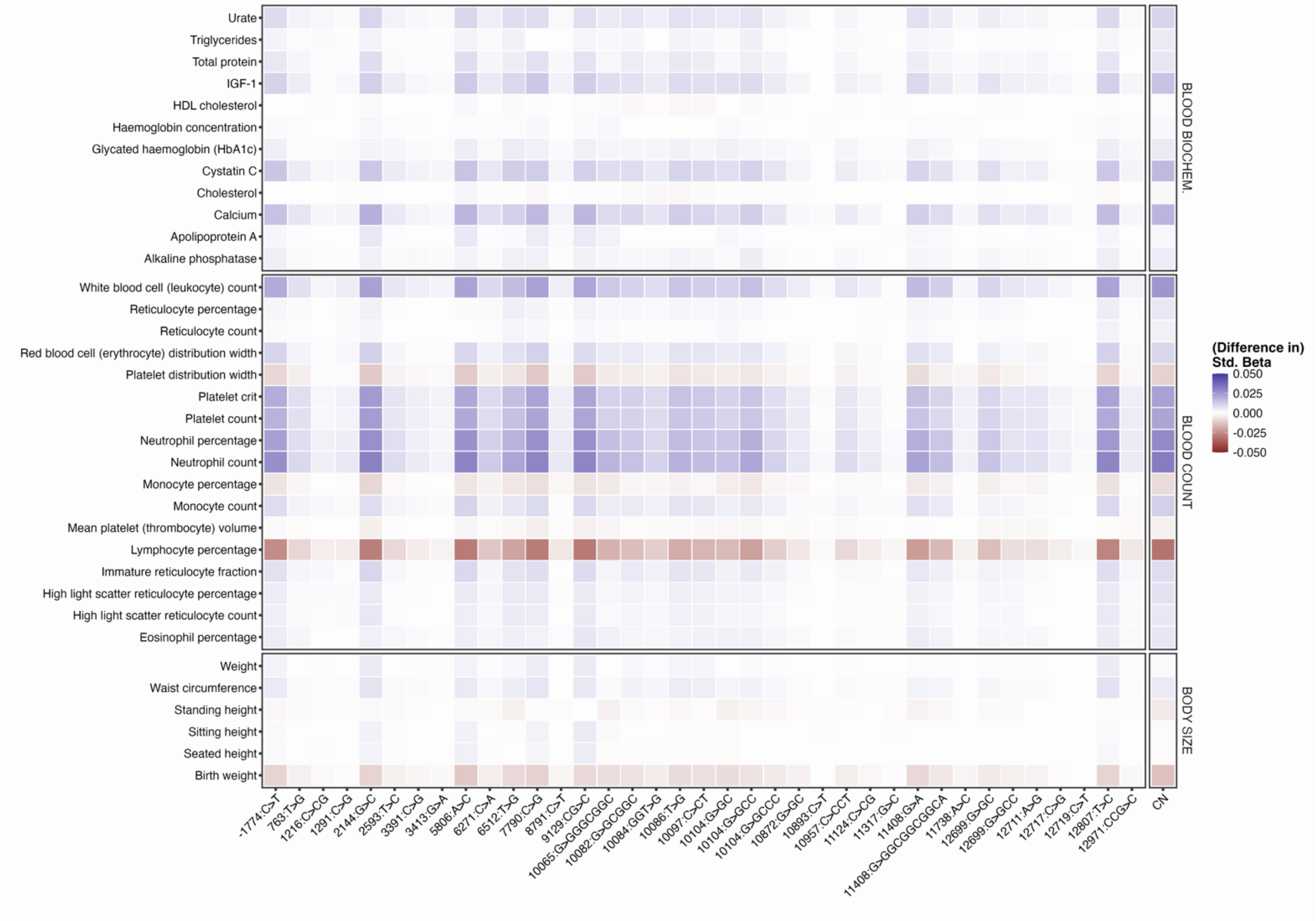

Figure S13: Differences in effect size between allele-specific CN and IGF in a selection of variants and traits, related to Figure 4. Heatmap of differences in effect sizes obtained from regression models of allele-specific CN and intragenomic variant frequency, for combinations of variants and phenotypes with variant-level associations included in **Fig. 3B** (left) compared with the effect sizes obtained for those same phenotypes on total rDNA CN associations (right).

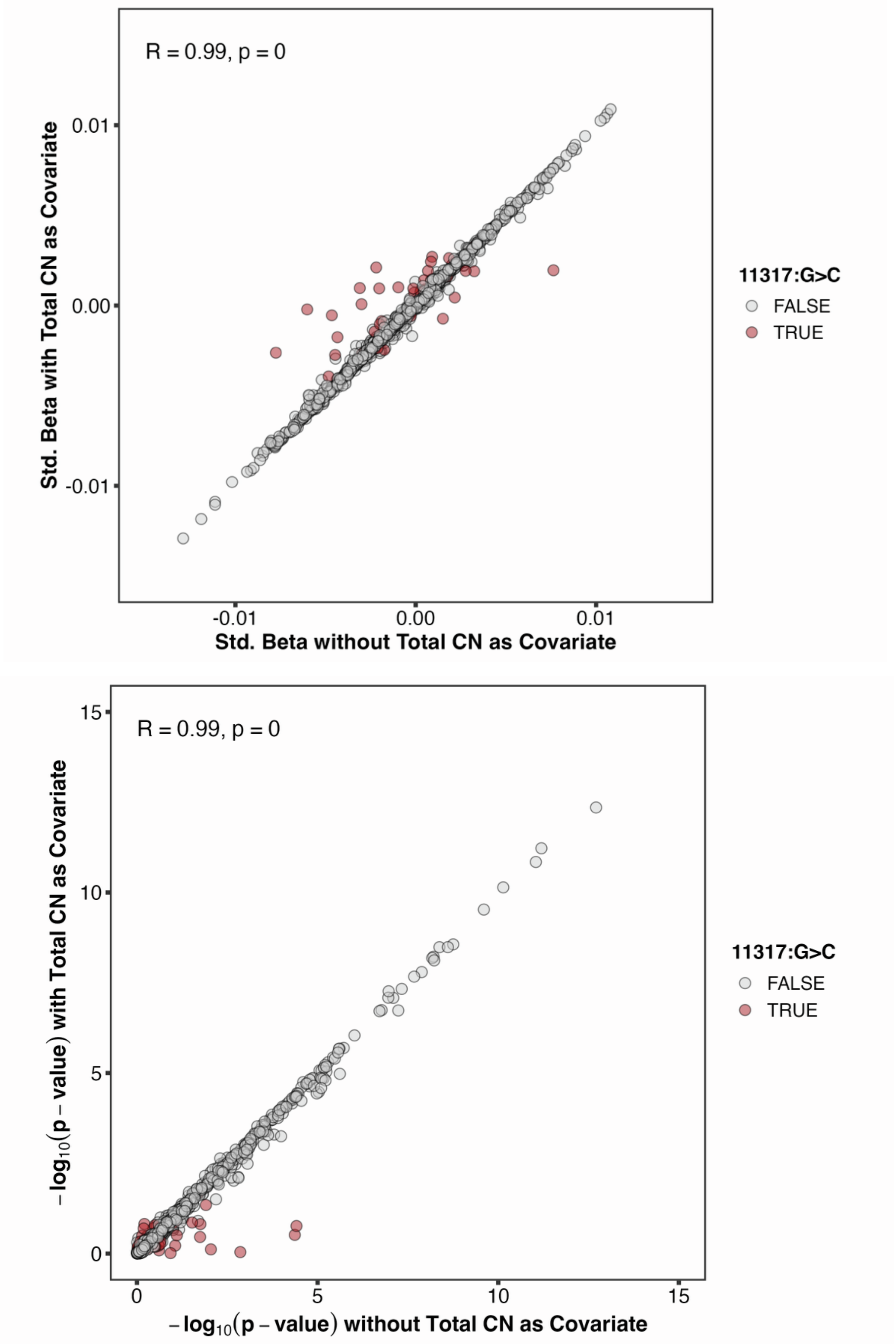

Figure S14: Impact of CN as covariate in IGF regression models, related to Figure 4. Comparison between effect sizes (Top) and significance levels (Bottom) obtained for all phenotypes and variant combinations in **Fig. 3B** (Pearson's R, N = 1295) with and without including total rDNA copy number as a covariate in the regression models. Points in red correspond with phenotypic associations of variant 11317:G>C.

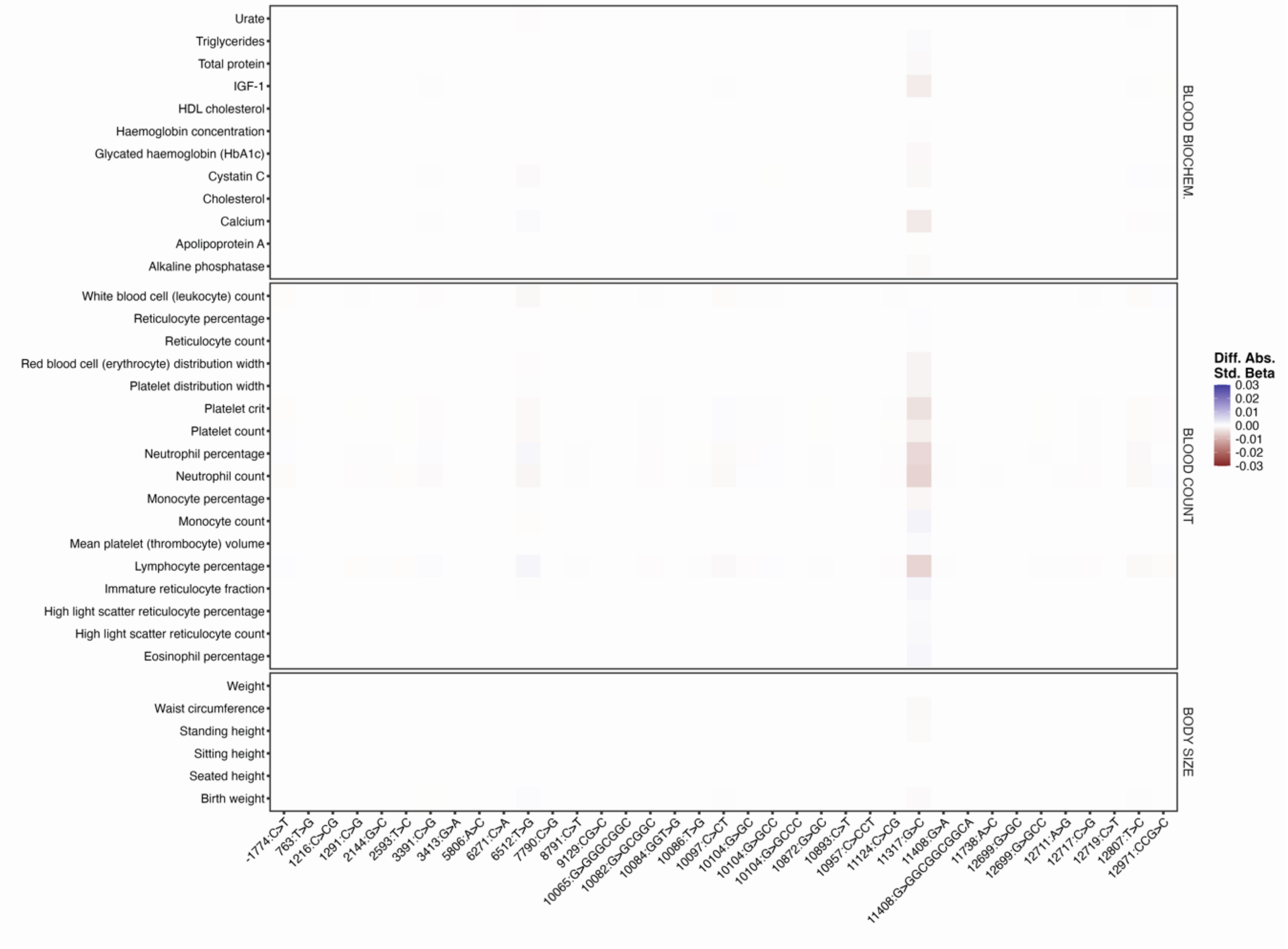

Figure S15: Differences in effect size in IGF regression models when including CN as covariate, related to Figure 4. Heatmap of differences in absolute effect sizes obtained from regression models of variant frequency with and without total rDNA CN as covariate, for combinations of variants and phenotypes with variant-level associations included in **Fig. 3B**.

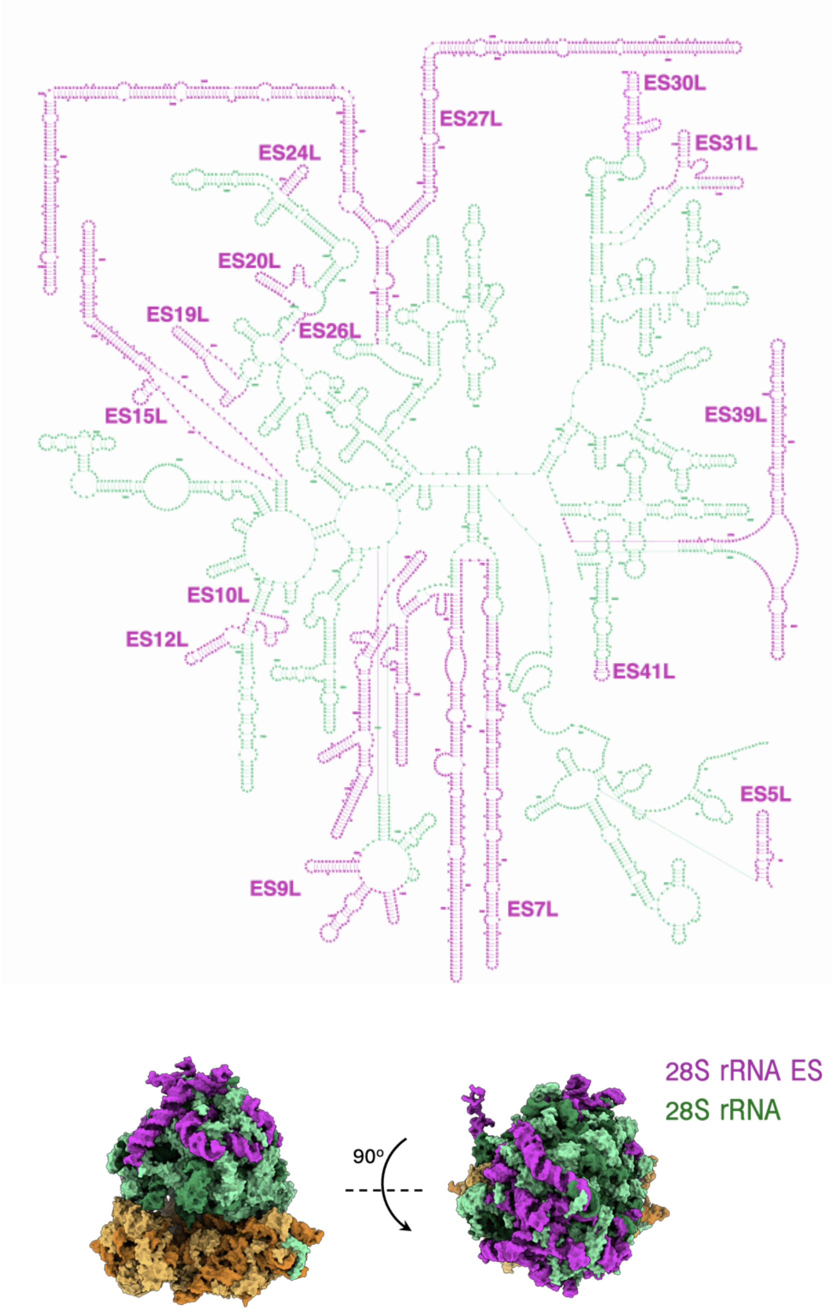

Figure S16: Secondary and tertiary structure of 28S Expansion Segments, related to Figure 5. Modelling of the location of 28S expansion segments (ES) both within the 2D rRNA (Top) and the 3D ribosome (Bottom) structures. Ribosome models generated from PDB accession 8QOI[71].

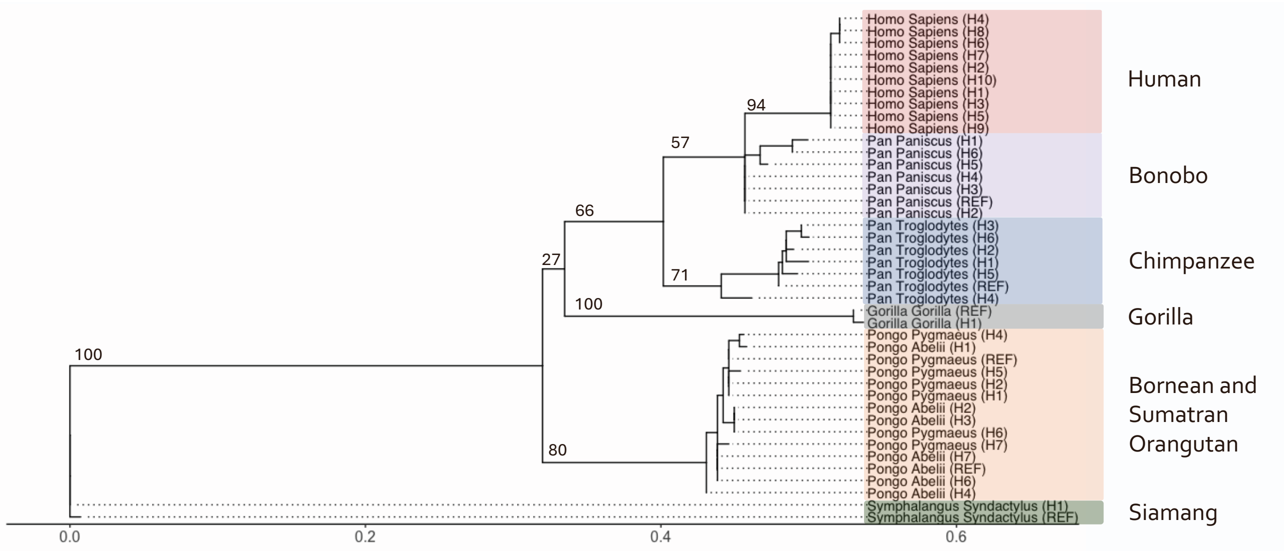

Figure S17: Phylogenetic tree of Expansion Segment 15L across apes, related to Figure 5. Phylogenetic tree comparing the 10 most frequent combinations of ES15L variants in the UKB with morphs for the equivalent region detected in various primates. Numbers in branches indicate the corresponding bootstrap support values. The x axis represents the estimated number of substitutions per base between sequences, reflected in the branch lengths.

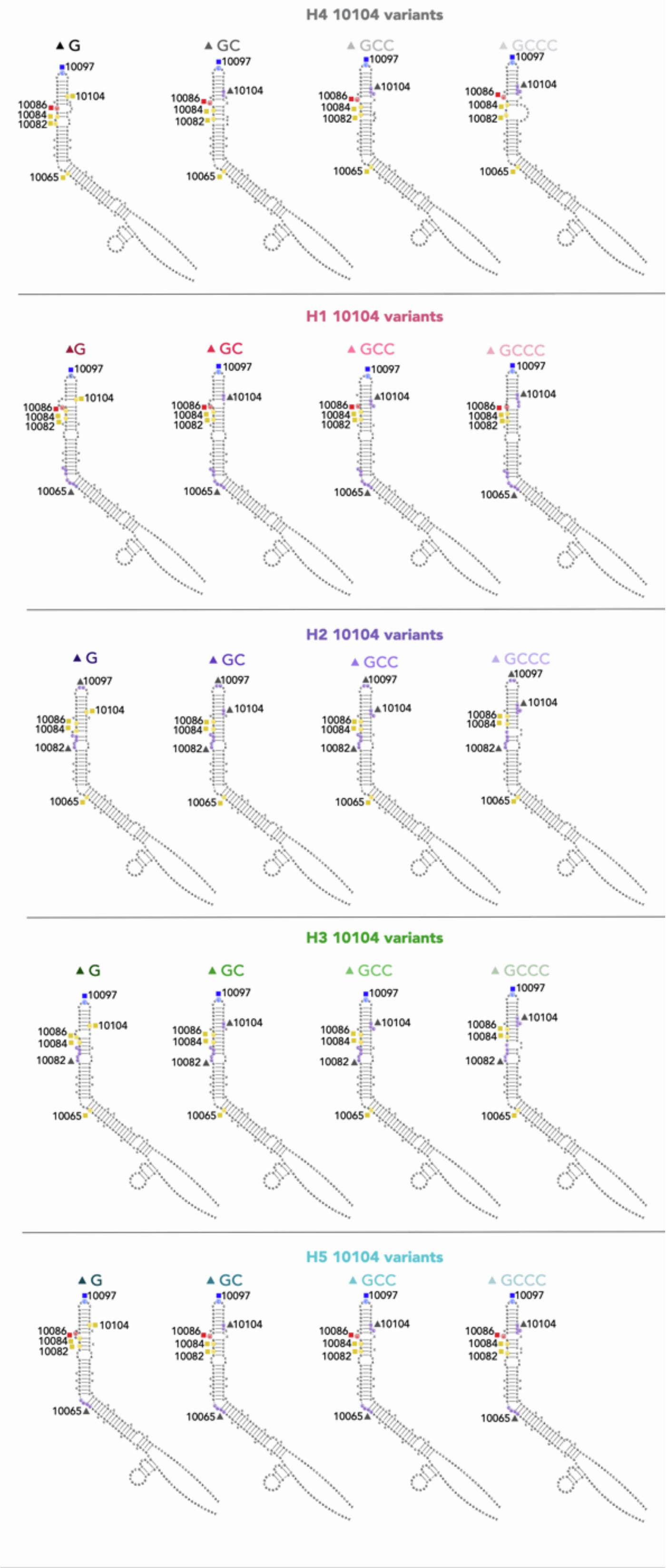

Figure S18. Predicted secondary structures arising from the variants at position 10104, related to Figure 5. Symbols and colours indicate variants as in **Fig. 5B**, including ES15L haplotypes H1-5.

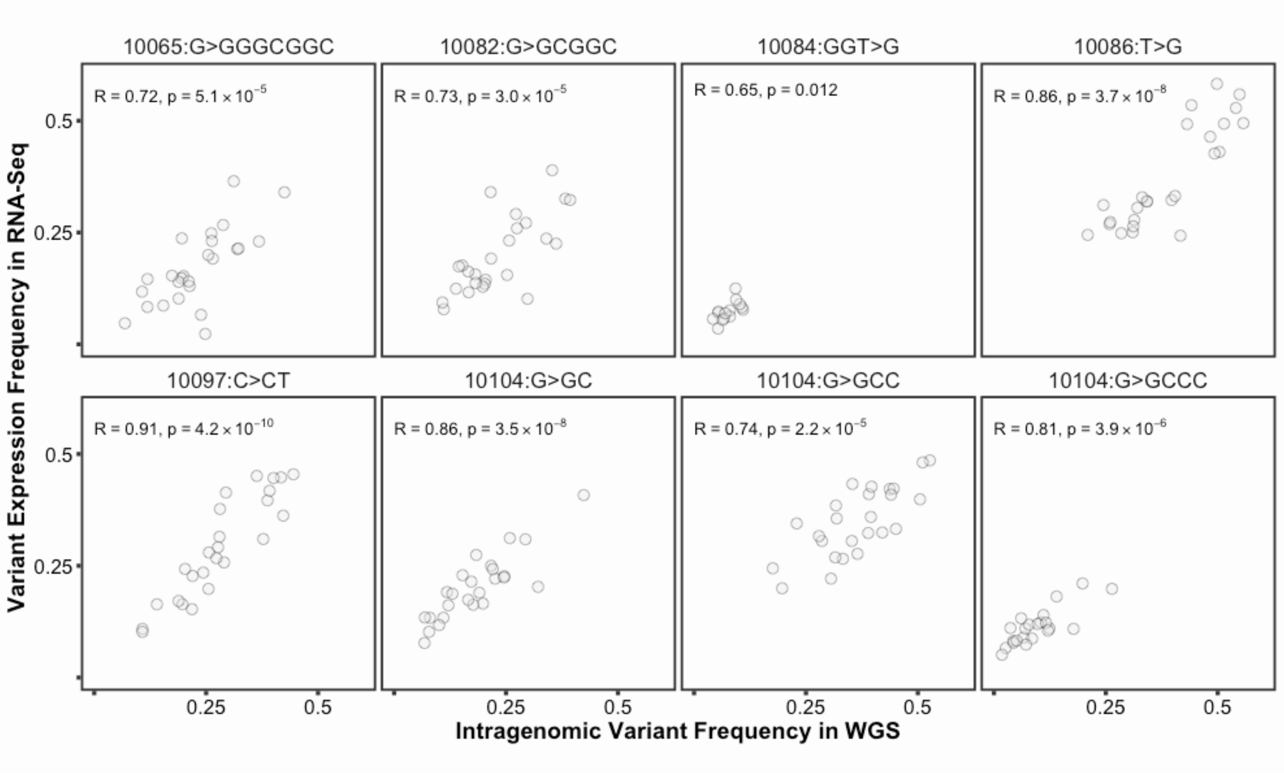

Figure S19: ES15L variant frequencies in DNA and RNA, related to Figure 5. Comparison between variant frequencies obtained on publicly-available WGS and RNA-seq data[42] of GBR participants of the 1000 Genomes Project, for UKB phenotype-associated variants in ES15L (Pearson's R, N = 25).

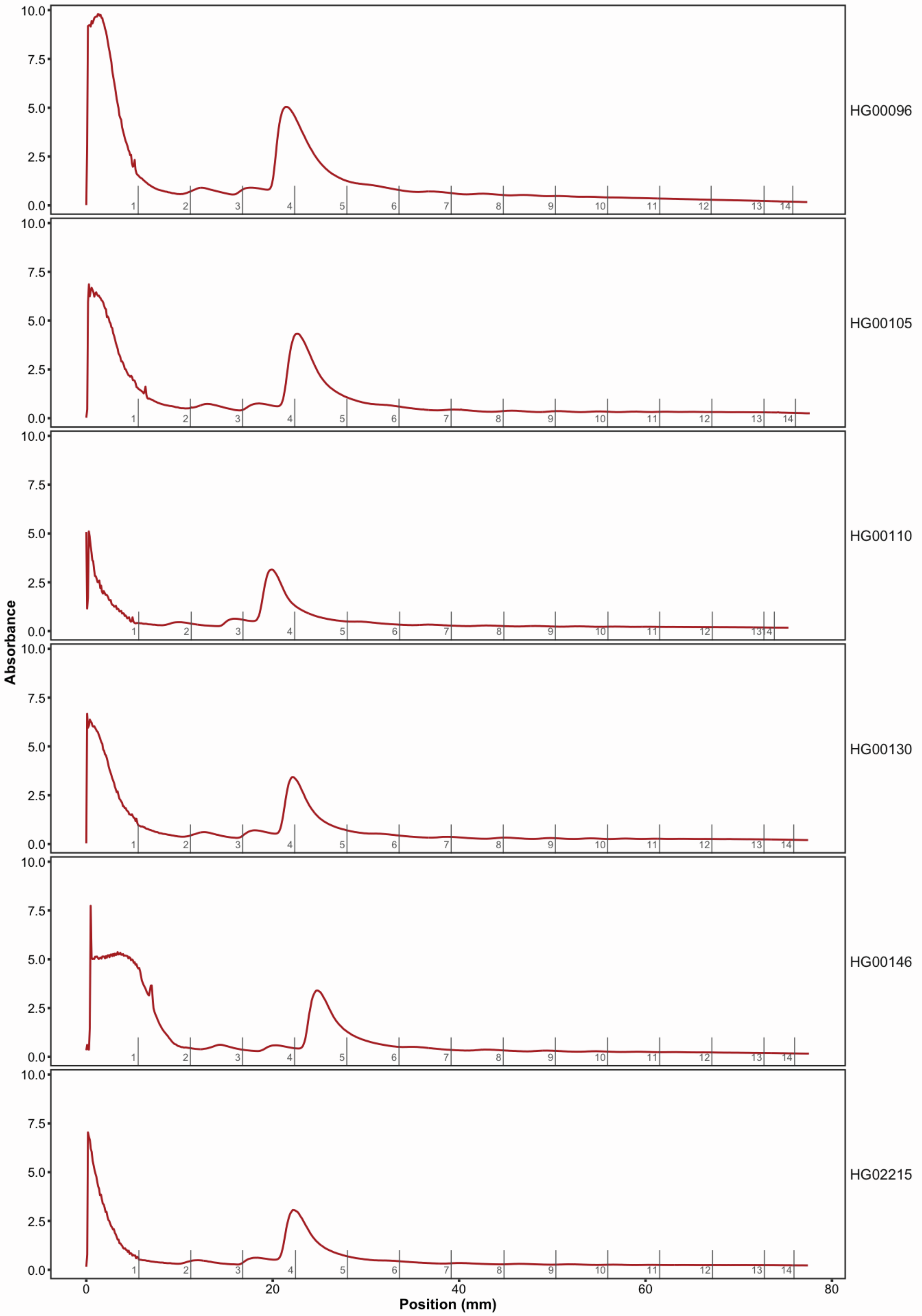

Figure S20: Sucrose absorbance profiles from human LCLs, related to Figure 5. Sucrose absorbance profiles for the six 1kGP GPR participants for which we generated Input RNA and Polysome-seq data.

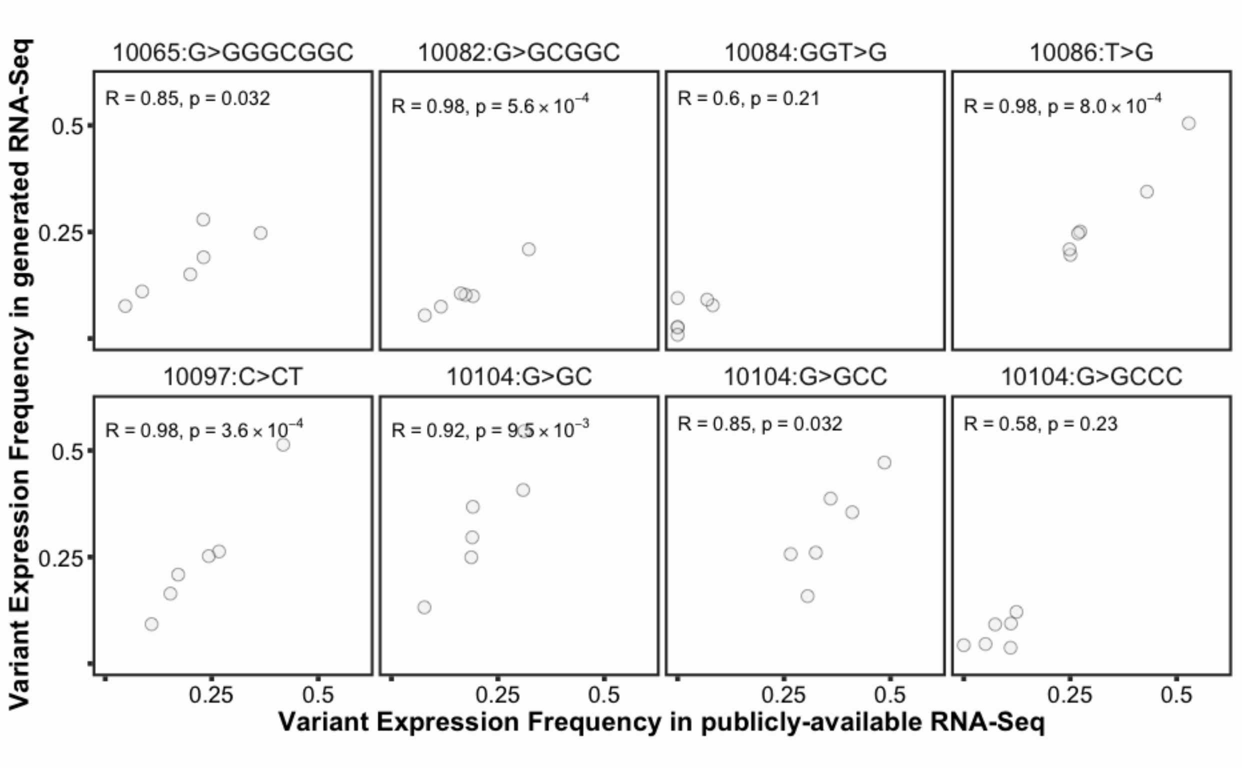

Figure S21: ES15L variant frequencies RNA-seq datasets, related to Figure 5. Comparison between variant frequencies obtained on publicly-available[42] and our own Input RNA-Seq of GBR participants of the 1000 Genomes Project, for UKB phenotype-associated variants in ES15L (Pearson's R, N = 6).

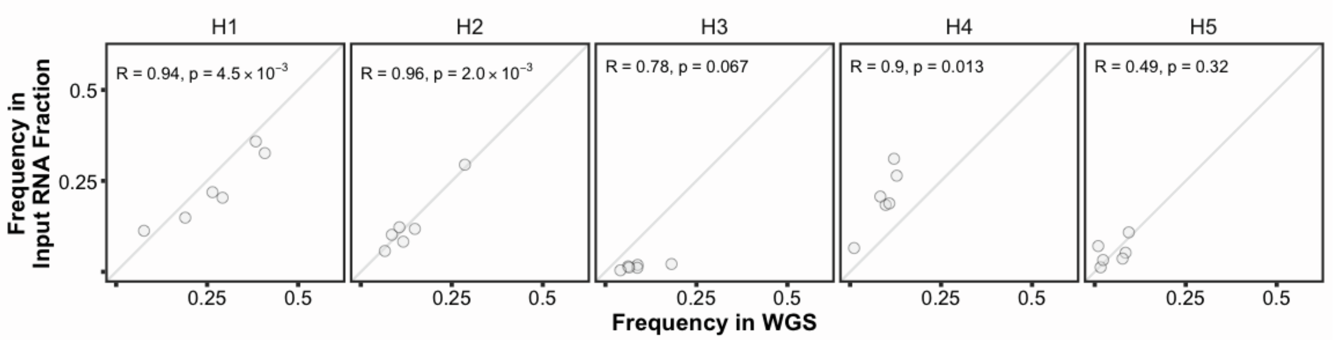

Figure S22: ES15L variant combination frequencies in DNA and RNA, related to Figure 5. Comparison between frequencies of the five most common combinations of ES15L variants (see **Fig 5B**) obtained on DNA and RNA data from GBR participants of the 1000 Genomes Project, for UKB phenotype-associated variants in the region (Pearson's R, N = 6).

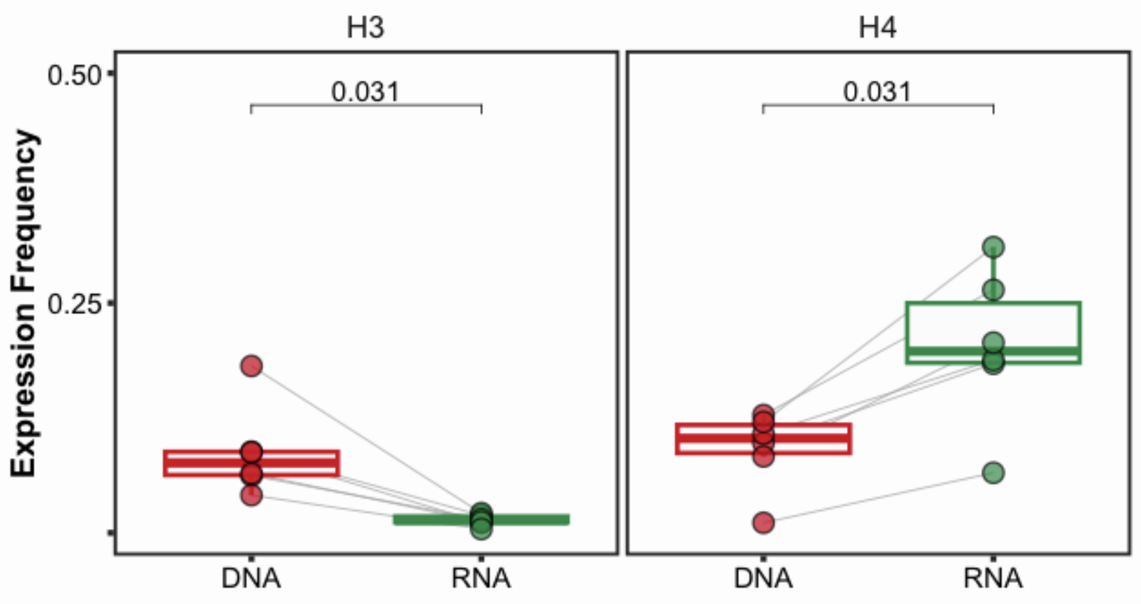

Figure S23: Prevalence of two ES15L variant combinations in DNA and RNA, related to Figure 5. Change in the prevalence of ES15L variant combinations H3 and H4 between WGS and mRNA data in 6 GBR 1kGP participants. p-values in paired Wilcoxon rank test for the difference of means indicated.

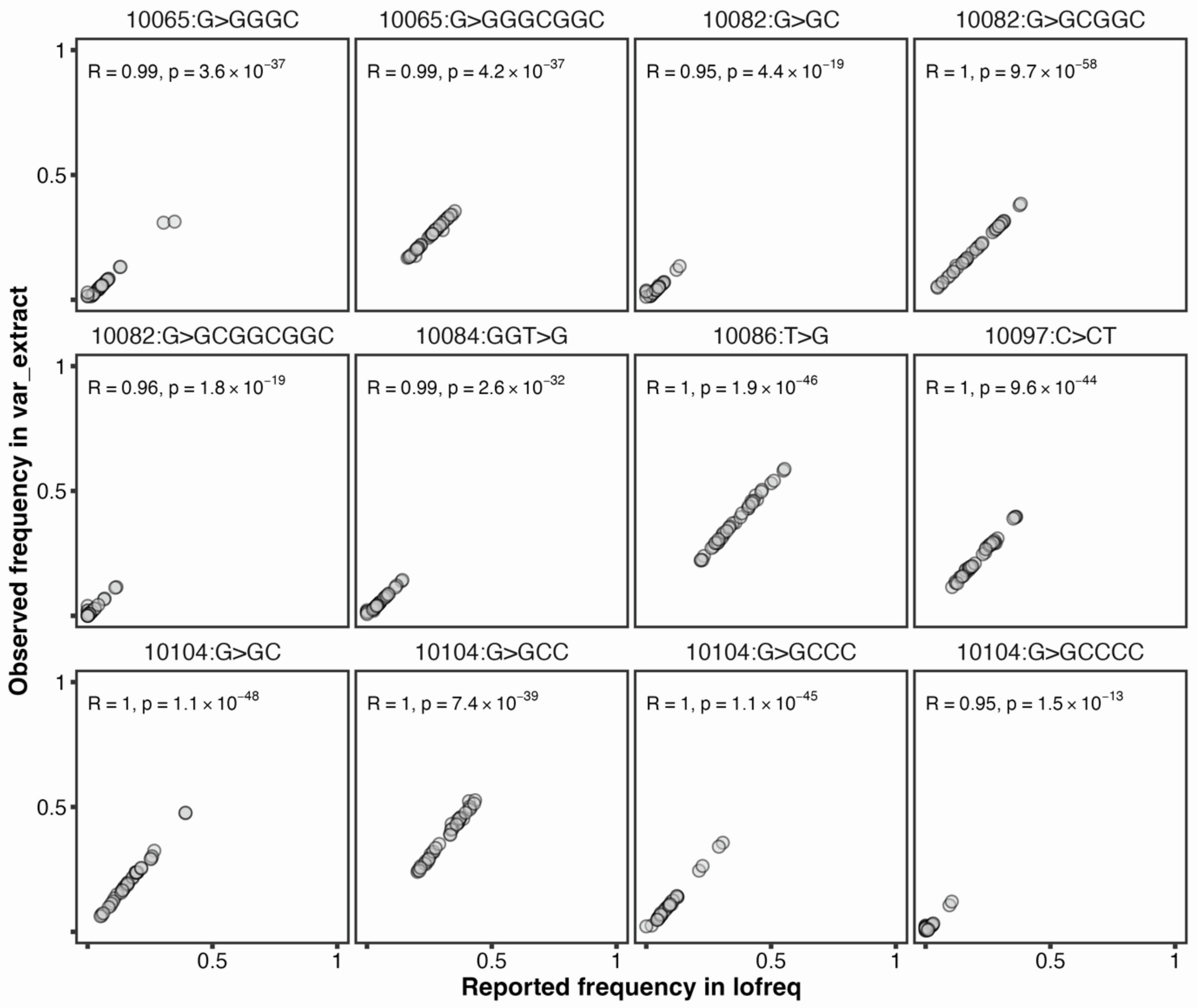

Figure S24: Validation of `var_extract` IGF estimates, related to STAR Methods. Comparison between ES15L variant frequencies obtained from `lofreq` and those derived from the per-read variant calls from the in-house `var_extract` script on MZ twin pairs included in the second deCODE WGS release (Pearson's R, N = 21 twin pairs).
